# Knowledge, attitude and practice of puerperal adolescents after an educational intervention on the mini contraceptive pill *

**DOI:** 10.1590/1518-8345.7227.4350

**Published:** 2024-10-25

**Authors:** Claudionete Abreu Costa, Ana Karina Bezerra Pinheiro, Lena Maria Barros Fonseca, Tatiane Gomes Guedes, Rita da Graça Carvalhal Frazão Corrêa, Adriana Gomes Nogueira Ferreira

**Affiliations:** ^1^ Universidade Federal do Maranhão, São Luís, MA, Brazil.; ^2^ Universidade Federal do Ceará, Departamento de Enfermagem, Fortaleza, CE, Brazil.; ^3^ Universidade Federal de Pernambuco, Recife, PE, Brazil.

**Keywords:** Adolescent, Postpartum Period, Hormonal Contraception, Breast Feeding, Nursing, Educational Technology

## Abstract

to evaluate the effectiveness of an educational intervention on the knowledge, attitude and practice of adolescent puerperal women regarding the mini-pill as a method of contraception in the postpartum period.

a quasi-experimental study with pre- and post-tests carried out with 139 puerperal adolescents in four public maternity hospitals, using an instrument with sociodemographic/gyneco-obstetric questions and a targeted survey called Knowledge, Attitude and Practice. The following were used in the analysis: Statistical Package for the Social Sciences software, version 20.1 for Windows; 5% significance level; 95% confidence interval; Shapiro-Wilk test to test data normality and paired t-test.

the average scores for the Knowledge, Attitude and Practice instrument were inadequate before the educational intervention; after the intervention, they remained inadequate, but with a slight improvement; the attitude was markedly inadequate. This instrument, before the intervention, was associated with age, education, number of pregnancies and having had prenatal care; after the intervention, in addition to these, it was associated with other variables, such as place of residence, being in school and having used contraception. The three domains were associated before and after the intervention.

the educational intervention, mediated by the nurse, was effective in promoting the knowledge, attitude and practice of puerperal adolescents in the proper use of the mini-pill as postpartum contraception.

## Highlights

**(1)** Statistically significant association between sociodemographic and gynecological-obstetric variables.

**(2)** The puerperal women had inadequate knowledge, attitudes and practices about the mini-pill.

**(3)** After two months, there was an increase in the puerperal women’s knowledge and practice.

**(4)** The average Knowledge, Attitude and Practice scores before the intervention and after 60 days remained inadequate.

**(5)** The technology used in the educational intervention is effective in promoting the Knowledge, Attitude and Practice domains.

## Introduction

 According to the Pan American Health Organization (PAHO), Brazil had one of the highest adolescent pregnancy rates in Latin America in 2016, with 68.4 live births per 1,000 girls aged 15 to 19 ^(^ 2018 ^)^ . Although the percentage of adolescent pregnancies has decreased over the years in all regions of the world (albeit unevenly), the recurrence of pregnancies remains stable - at around 20% ^(^ 2020 ^-^ 2020 ^)^ . 

 In 2010, almost 20% of all births in Brazil were to adolescents, while in 2019 the proportion was 14.72%. The biggest decrease was in the 15-19 age group. The youngest adolescents, aged 10 to 14, showed proportions below 1% and a more discreet downward trend ^(^ 2018 ^)^ . 

 A geoprocessing study that analyzed the spatial variation of adolescent pregnancy in Brazil showed that the North, Midwest and Northeast regions had higher median rates of adolescent fertility among women aged 15 to 19 ^(^ 2021 ^)^ . In the Northeast Region, in 2020, Maranhão was responsible for 23,132 births to adolescent mothers, and of these, the municipality of São Luís registered 1,932 births and São José de Ribamar 498 births to mothers aged between 10 and 19 years old ^(^ 2023 ^)^ . 

 The lack of or poor practice of reproductive planning in adolescence has been a strong ally in delaying the reduction in the percentage of adolescent pregnancies and the instability of repeat pregnancies ^(^ 2020 ^)^ , however, the difficulties related to adherence, in terms of the use of contraceptive methods by adolescent mothers, represent some of the factors that keep pregnancy rates high, especially recidivism ^(^ 2020 ^,^ 2022 ^-^ 2023 ^)^ . 

 With regard to specific knowledge about hormonal contraceptive methods, the adolescents recognize that the contraceptive pill does not prevent Sexually Transmitted Infections (STIs), but they do not know how to distinguish the action of oral contraceptives, including the mini-pill. Mini-pills inhibit pregnancy exclusively through progestational effects and are recommended while breastfeeding ^(^ 2021 ^-^ 2021 ^)^ . 

 Contraceptive counseling is an important strategy, and the various technologies (workshops, educational booklets, albums, telephones) should be used to facilitate the sharing of information and experiences, providing the opportunity for knowledge, for adherence to the conscious contraceptive method ^(^ 2020 ^)^ . Nurses can use them creatively in education and health to achieve their goals of adopting healthy behaviors, identifying deficiencies and weaknesses and contributing to knowledge and healthy practices ^(^ 2022 ^)^ . 

 Among educational technologies, the serial album stands out, made up of sequential pages with illustrations, maps, single and direct messages, used in lectures, meetings and to support classes. This resource has proved to be an excellent way of disseminating knowledge to all audiences (young adults and adolescents), and is very suitable for carrying out educational activities without jeopardizing the interaction between the educator and their audience ^(^ 2020 ^)^ . 

It is therefore important to carry out studies that test the effectiveness of educational technologies, such as the serialized album discussed here, and thus guarantee safe information in order to have a positive impact on the knowledge, attitude and contraceptive practice of puerperal adolescents.

The aim of this study was to evaluate the effectiveness of an educational intervention on the knowledge, attitude and practice of adolescent puerperal women regarding the mini-pill as a method of contraception in the postpartum period.

## Method

### Study design

 This is an evaluative study of the knowledge, attitude and practice (KAP) type ^(^ 2010 ^)^ , with a quasi-experimental design with a single group, of the before and after type ^(^ 2019 ^)^ , cross-sectional ^(^ 2015 ^)^ , using the serial album. 

### Period and location

Data collection took place between April and July 2022 in four public maternity hospitals, three in the municipality of São Luís (MA) and one in the municipality of São José de Ribamar (MA). Two maternity hospitals were characterized as high-complexity hospitals for high-risk pregnant women and the other two as regular-risk maternity hospitals. This option made it possible to identify the PAC of puerperal women cared for in maternity hospitals with different profiles.

### Population and sample

We chose to include adolescents aged between 10 and 19, according to the World Health Organization’s (WHO) definition, which defines adolescence as the second decade of life. These adolescents, regardless of the type of pregnancy or birth, were admitted to the rooming-in unit. Puerperal adolescents who had suffered any kind of violence during pregnancy and childbirth and who were in an isolation area due to an infectious disease were not included. The sample, made up of 139 adolescent puerperae who answered the pre- and post-test after taking part in the educational intervention, was obtained by consecutive non-probabilistic sampling. The pre-test was carried out with puerperal women at least 12 hours postpartum and the post-test at up to 6 weeks postpartum.

The study was organized into four phases:

- Phase I - organization of the serial album, with content selected from the Ministry of Health’s Primary Care Notebook on sexual and reproductive health and the Advice Cards on Contraceptive Methods for adolescents, referring to the use of the mini-pill. The serialized album is an educational material based on scientific knowledge used as a tool in health education and guidance activities. After adaptation, the album was tested with a group of puerperal adolescents, who were not included in the research, with the aim of evaluating its application, allowing for adjustments that would favor better understanding by the participants.
- Phase II - recruitment of participants and application of the pre-test, using an instrument with sociodemographic questions (related to age, residence, education) and gyneco-obstetric questions (about contraceptive use, number of pregnancies, prenatal care) and the KAP survey (questions about knowledge, attitudes and practices related to contraception through the use of the mini-pill).
- Phase III - application of the educational intervention using the serialized album and clarification of doubts about the pre-test questions.
- Phase IV - post-test, carried out using the cell phone as a tool to apply the KAP survey.

The serialized album consists of three pages, illustrated with female figures, including an adolescent puerperal woman talking to the nurse. The first sheet contains questions and answers relating to knowledge about the correct use of the mini-pill for postpartum contraception, so that the exchange of information is done in a playful way. On the second sheet, the dialog expresses the active role of the puerperal woman in making the decision to use the mini-pill correctly, as a way of receiving the first feedback on the knowledge acquired. The third sheet deals with the practice of this adolescent puerperal woman after the educational intervention carried out by the nurse, demonstrating her understanding of the subject and the practice of using the mini-pill properly. On this sheet of the album, only the adolescent puerperal woman appears as the protagonist of her choices, in other words, of her appropriate practices regarding the method.

### Data collection

For the data collection, which took place at two different times, the pre-test and the post-test, the researcher was assisted by duly trained fellows. The pre-test took place in person, during hospitalization in the rooming house, according to the suggested timing, so that visiting hours were avoided in order to prioritize individuality. During the first contact, the proposal was presented, as well as the dynamics of the educational intervention, the stages of data collection and clarification to improve knowledge about the correct use of the mini contraceptive pill, with a view to preventing the recurrence of an unplanned pregnancy.

 After the pre-test, the puerperae were given instructions for the subsequent post-test stage, using the cell phone up to 6 weeks after giving birth ^(^ 2016 ^)^ . 

Initially, the researcher presented the serial album to the puerperae and informed them that it contained the same questions they had already answered in the pre-test. The puerperae watched them carefully for 5 minutes, checking which answers they had got right or wrong. After the explanations, they asked questions. Orientation was carried out in sequence, from knowledge to practice, lasting 20 minutes when individualized and 40 minutes when it was possible for them to talk to the researcher.

In the second stage, the post-test was administered, including the KAP. To conduct this stage, a spreadsheet was organized with each participant’s details, including telephone contact, date of delivery and pre-test application. The phone call to the participants was followed by the identification of the researcher, the activity that was going to be carried out and a reminder of the pre-test that was carried out at the time of hospitalization, followed by the post-test questions (KAP survey). Up to three attempts were made when the call was not answered the first time. Each conversation lasted between 10 and 15 minutes. The answers were marked on a new instrument identified as the post-test, with the respective date, and recorded in the spreadsheet to organize the database.

### Instrument used

 The KAP survey instrument was used to assess the three dimensions (KAP) ^(^ 2019 ^)^ , applied at two points in time: before and after the educational intervention, to measure the main outcome relating to the three domains (KAP) ^(^ 2018 ^-^ 2018 ^)^ of adolescent postpartum women regarding the use of the mini contraceptive pill. 

Knowledge was considered adequate if the participant answered about the mini-pill: “the pill indicated for women who are breastfeeding is the mini-pill”; “if the woman experiences vomiting and/or diarrhea for more than 24 hours, she should use a condom until the next menstrual cycle”; “some medications interfere with the effect of the mini-pill”; “if the woman vomits within 1 hour of taking the pill, she needs to take another pill”; “the pill should be taken every day, at the same time”; “to start taking the pill, you need to consult a doctor and have a prescription”; “when a pack of pills is finished, the woman should start a new pack straight away, without a break”; “the mini-pill should not be started from the first day after giving birth”; taking the mini-pill can cause menstruation to change”. The instrument was considered adequate if it had seven to nine correct correlations and inadequate if it had less than seven correct correlations.

The attitude was considered adequate if the adolescent puerperal woman reported: “intending to use a contraceptive method and/or the mini-pill while breastfeeding and the pill afterwards”; “stating that she will consult a health professional to start using the mini-pill/pill”; “starting the mini-pill 6 weeks after giving birth”. The attitude was considered inadequate if the participant said she had no intention of using the mini-pill for any reason.

The practice was considered adequate if the adolescent puerperal woman stated that she would “only use the mini-pill after consulting a doctor and with a prescription”; “start taking the mini-pill after 6 weeks postpartum, taking it every day at the same time”; “if you forget to take a pill, take the forgotten pill as soon as you remember and the next pill at the usual time”; “when you finish taking a mini-pill, start a new one without a break”. The practice was considered inadequate if the adolescent said that she “won’t use the mini-pill when she starts sexual activities after giving birth, or starts taking it on her own”.

### Data analysis

For data analysis, descriptive statistics were applied with dependent variables (KAP) about the mini-pill and independent variables (sociodemographic and obstetric variables) to establish the types of activities carried out associated with the adoption of undesirable behavior. After collection, the data was coded and stored in an Excel spreadsheet, version 20.1. Initially, descriptive analyses were carried out (frequency, mean, standard deviation, minimum and maximum) of the variables, the results were presented and the statistical analysis was carried out using the Statistical Package for the Social Sciences software, version 20.1 for Windows, with a 5% significance level and 95% confidence interval. The Shapiro-Wilk test was used to test the normality of the data and the paired t-test was used to verify differences in KAP before the intervention and after the intervention, considering a p-value of less than or equal to 0.05.

### Ethical aspects

 All the ethical aspects relating to research with human beings were respected, as determined by Resolution 466/12 of the Ministry of Health and its complementary resolutions ^(^ 2013 ^)^ . The study was approved by the Research Ethics Committee of the University Hospital of the Federal University of Maranhão (protocol 4.988.517). 

## Results

During data collection, 151 puerperal adolescents took the pre-test, however, at the post-test stage, 12 participants were lost, adding up to 139 adolescent puerperae in the total sample.

 The data before the intervention showed that knowledge, attitude and practice were considered adequate, with 33.81%, 15.82% and 19.42%, respectively, with an average score between the three domains of 32 for “adequate” ( Table 1 ). 

**Table 1:** - Knowledge, attitude and practice of adolescent puerperae before the educational intervention. São Luís and São José de Ribamar, MA, Brazil, 2023

KAP*	**n**	**SD** ^†^	**Minimum**	**Maximum**	**Score**		**Total**
					**Adequate** **n (%)**	**Inadequate** **n (%)**	
Knowledge	139	2.68	-	29	47 (33.81)	92 (66.18)	139
Attitude	139	3.14	01	28	22 (15.82)	117 (84.17)	139
Practice	139	4.97	-	31	27 (19.42)	112 (80.57)	139
Mean KAP*					32	107	139

* KAP = Knowledge, Attitude and Practice

^†^ SD = Standard Deviation

 After the intervention, there was an increase in the three KAP domains. There was a decrease in the average from adequate to 51. However, when the attitude domain was analyzed separately, there was an increase in the number of participants with inadequate attitudes ( Table 2 ). 

 When KAP was associated with socio-demographic and gynecological-obstetric variables before the intervention, there was a statistically significant association for the variables age, schooling, number of pregnancies and prenatal care (p=0.05) ( Table 3 ). 

 In the association of adequate KAP with the socio-demographic and gynecological-obstetric variables after the intervention, there was a statistically significant association between the variables age, education, number of pregnancies, prenatal care, place of residence and education. As for the variable “used some contraceptive method”, there was only an association with the attitude domain variables “age, schooling, number of pregnancies, prenatal care, place of residence and schooling”. As for the variable “used some contraceptive method”, there was only an association with the attitude domain ( Table 4 ). 

 In the association between the knowledge, attitude and practice domains before the intervention, there was a statistically significant association between attitude and practice (p<0.05). After the intervention, there was a significant association between the three domains: practice with knowledge (p<0.05) and attitude (p= 0.05) ( Table 5 ). 

**Table 2:** - Knowledge, attitude and practice of adolescent puerperae after educational intervention. São Luís and São José de Ribamar, MA, Brazil, 2023

KAP*	**n**	**SD** ^†^	**Minimum**	**Maximum**	**Score**		**Total**
					**Adequate** **n (%)**	**Inadequate** **n (%)**	
Knowledge	139	2.59	-	37	90 (64.74)	49 (35.25)	139
Attitude	139	3.18	-	35	13 (9.35)	126 (90.64)	139
Practice	139	3.26	-	36	51 (36.69)	88 (63.30)	139
Mean KAP					51	88	139

* KAP = Knowledge, Attitude and Practice

^†^ SD = Standard Deviation

**Table 3:** - Association of knowledge, attitude and practice with sociodemographic and gynecological-obstetric variables of adolescent puerperae before the educational intervention, by correlation analysis. São Luís and São José de Ribamar, MA, Brazil, 2023

**Variables**	**Total**	**Proper knowledge**		**Proper Attitude**		**Proper Practice**	
		**n (%)**	**p-value**	**n (%)**	**p-value**	**n (%)**	**p-value**
**Age (years** )							
12-13	28	2 (7.1)		2 (7.1)		2 (7.1)	
14-15	51	1 (1.9)		1 (1.9)		2 (3.9)	
16-17	52	6 (11.5)		-		4 (7.6)	
18-19	8	6 (75.0)		6 (75.0)		5 (62.5)	
**Residence area**							
Rural	61	5 (8.19)	0.31	4 (6.5)	0.23	5 (8.1)	0.11
Urban	78	8 (10.25)		7 (10.2)		9 (11.5)	
**Education**							
Elementary school complete	2	1 (50.0)	0.05	-	0.05	-	0.05
Elementary school incomplete	4	3 (75.0)		2 (50.0)		-	
Elementary school II complete	2	-		-		-	
Elementary II incomplete	58	15 (25.8)		10 (17.2)		8 (13.7)	
High school complete	31	13 (41.9)		7 (22.5)		5 (16.1)	
High school incomplete	42	12 (28.5)		9 (21.4)		6 (14.2)	
**Studying**							
Yes	39	12 (30.8)	0.01	9 (23.1)	0.18	9 (23.1)	0.14
No	100	5 (5.0)		4 (4.0)		7 (7.0)	
**Contraceptive method**							
Yes	14	5 (35.7)	0.33	6 (42.9)	0.05	6 (42.9)	0.33
No	125	12 (9.6)		11 (8.8)		21 (16.8)	
**Pregnancy**							
1	98	18 (18.4)	0.05	21 (21.4)	0.05	15 (15.3)	0.05
2	27	13 (48.1)		15 (55.6)		12 (44.4)	
3 or more	14	3 (42.2)		3 (33.3)		1 (11.1)	
**Had prenatal care**							
Yes	116	29 (25.0)	0.05	20 (17.2)	0.05	22 (19.0)	0.05
No	23	8 (34.7)		5 (21.7)		7 (30.4)	

**Table 4:** - Association of knowledge, attitude and practice with sociodemographic and gyneco-obstetric variables of adolescent puerperae after educational intervention, by correlation analysis. São Luís and São José de Ribamar, MA, Brazil, 2023

**Variables**	**Total**	**Proper knowledge**		**Proper Attitude**		**Proper Practice**	
		**n (%)**	**p-value**	**n (%)**	**p-value**	**n (%)**	**p-value**
**Age (years)**							
12-13	28	4 (14.3)		4 (14.3)		4 (14.3)	
14-15	51	3 (5.8)		3 (5.8)		4 (7.8)	
16-17	52	10 (19.2)		2 (3.8)		6 (11.5)	
18-19	8	3 (37.5)		2 (25.0)		1 (12.5)	
**Residence area**							
Rural	61	7 (11.5)	0.05	6 (9.8)	0.01	7 (11.5)	0.01
Urban	78	10 (12.8)		9 (11.5)		11 (14.1)	
**Education**							
Elementary school complete	2	-	0.05	-	0.05	-	0.05
Elementary school incomplete	4	2 (50.0)		2 (50.0)		2 (50.0)	
Elementary school II complete	2	-		-		-	
Elementary II incomplete	58	17 (29.3)		12 (20.7)		10 (17.2)	
High school complete	31	15 (48.4)		9 (29.0)		7 (22.6)	
High school incomplete	42	14 (33.3)		11 (26.2)		8 (19.0)	
**Studying**							
Yes	39	14 (35.9)	0.04	11 (28.2)	0.04	9 (23.1)	0.05
No	100	7 (7.0)		6 (6.0)		7 (7.0)	
**Contraceptive method**							
Yes	14	6 (42.9)	0.31	7 (50.0)	0.04	7 (50.0)	0.17
No	125	13 (10.4)		12 (9.6)		22 (17.6)	
**Pregnancy**							
1	98	20 (20.4)	0.05	23 (23.5)	0.05	17 (17.3)	0.05
2	27	15 (55.6)		17 (63.0)		14 (51.9)	
3 or more	14	7 (50.0)		7 (50.0)		5 (35.7)	
**Had prenatal care**							
Yes	116	31 (26.7)	0.05	35 (30.1)	0.05	24 (20.7)	0.05
No	23	10 (43.4)		7 (30.4)		9 (39.1)	

**Table 5:** - Association between the knowledge, attitude and practice domains of adolescent puerperae before and after the educational intervention. São Luís and São José de Ribamar, MA, Brazil, 2023

	**Adequate** **n (%)**	**Inadequate** **n (%)**	**P-value**
**Practice before**			
Knowledge			
Adequate	1 (0.71)	11 (7.91)	0.12
Inadequate	3 (2.15)	26 (19.07)	
**Attitude**			
Adequate	1 (0.71)	15 (10.79)	0.04
Inadequate	2 (1.43)	36 (25.89)	
**Practice after**			
**Knowledge**			
Adequate	21 (15.10)	14 (10.07)	0.04
Inadequate	15 (10.79)	29 (20.86)	
**Attitude**			
Adequate	1 (0.71)	10 (7.19)	0.05
Inadequate	5 (3.59)	17 (12.23)	

## Discussion

 The puerperium is an opportune moment in the pregnancy-puerperium cycle to prevent, identify and treat alterations that compromise women’s health ^(^ 2022 ^)^ , such as the recurrence of unplanned adolescent pregnancies. 

 The present study showed that the distribution of scores related to the means of the knowledge, attitudes and practice domains regarding the use of the mini-pill before the educational intervention were low, showing inadequate knowledge of contraceptive methods and the vulnerability of adolescent puerperae, including the mini-pill as a method of preventing recurrence of pregnancy in the postpartum period. A qualitative study carried out in Thailand with adolescents on the recurrence of pregnancy found that the reasons were lack of contraceptives, lack of knowledge about methods and lack of awareness, which was characterized by knowing how to use them but not using them and intending to use them but not using them ^(^ 2023 ^)^ . 

 A national study carried out in Pernambuco also highlights the male condom as the best known, followed by oral contraceptives, but the participants did not know which type of pill to use, when and how to use them ^(^ 2022 ^)^ , coinciding with this study and demonstrating the fragility of these adolescents’ attitude towards putting their knowledge into practice due to a lack of information. 

 After the educational intervention, the adolescent puerperal woman’s attitude domain increased its average inadequacy score compared to the other domains, which remained inadequate, but showed improvements. It is believed that the intervention increased the participants’ knowledge about the proper use of the mini-pill, but it was not enough to change their attitude towards proper practice. It is important to complement care with educational interventions in order to improve knowledge, as observed in a study that used educational videos in Thailand, as a complement to the traditional method of counseling in the physician’s office, which significantly increased knowledge about the contraceptive method and, consequently, contributed to the preference for the methods presented ^(^ 2022 ^)^ . 

 When analyzing the association between sociodemographic variables and the adequate knowledge, attitude and practice of puerperal women before the educational intervention, it was found that the variables age and schooling showed a significant association in all three domains, as did the variable studying in the knowledge domain, demonstrating that being older and studying are indicators that favor the participants’ ability to understand and improve their knowledge, attitude and adequate practice. After the intervention, it was found that, in addition to age and schooling, other variables studied, such as place of residence, had significant associations in all three domains. In this context, the use of the serialized album in printed form, mediated by the nurse in the interactional relationship with the participants, was certainly very important, configuring an educational technology that serves to facilitate the acquisition of the KAP of adolescent puerperae on the use of the contraceptive method in question ^(^ 2020 ^)^ . 

 Research into contraceptive knowledge, attitudes and practices among adolescents revealed gaps that need attention to prevent unplanned adolescent pregnancies, which often occur in the postpartum period and can happen in the late puerperium (11 ^th^ to 45 ^th^ day) ^(^ 2023 ^)^ . The importance of advising parents about contraception before they start having sex is emphasized, as well as the role of health professionals in proactively discussing contraception through individualized and confidential communication ^(^ 2023 ^)^ . 

 National and international studies have shown an association between the recurrence of adolescent pregnancy and the socioeconomic and demographic characteristics of the population; single women; low income; inadequate schooling for their age and difficulty in accessing reproductive planning, especially in the postpartum period, reiterating the social inequalities that exist among the population. In this context, the female population’s unequal access to reproductive planning stands out ^(^ 2022 ^)^ . It should be noted that adolescent pregnancy is associated with an increase in maternal and fetal complications and is also responsible for an increase in the incidence of unsafe abortions and maternal mortality. It is therefore important to raise awareness of effective methods of postpartum contraception to prevent pregnancy at this stage of life ^(^ 2022 ^)^ . 

In the analysis of the association between the gynecological and obstetric variables and the puerperal women’s adequate knowledge, attitude and practice before the educational intervention, it was found that the variables number of pregnancies and having had prenatal care showed a significant association in all three domains. After the intervention, in addition to the two variables mentioned, the use of a contraceptive method had a significant association in the attitude domain. This result shows that the educational intervention was fruitful in terms of enabling people to understand the method and favored adherence to the practice of using the mini-pill properly.

 The results of a study carried out with adolescents and young women in Guinea revealed individual and community factors associated with the unmet need for contraception. The authors suggest that, in addition to sex education and awareness campaigns through the media and places where women gather, individual counseling in health services should be better targeted at this audience, taking into account their individual and contextual characteristics ^(^ 2023 ^)^ . 

 These indicators, combined with socio-economic factors, probably had a positive influence on awakening puerperal women to improve their knowledge, especially their attitude towards the proper use of the mini-pill as a contraceptive method in the postpartum period ^(^ 2019 ^)^ . 

 When analyzing the association between the three domains, it was found that the association is significant in the relationship between attitude and appropriate practice, and it is possible to infer that the participant’s decision to practice the method presented is related to the knowledge acquired previously and is based on their own needs to use some preventive method. After the educational intervention, the practice had a significant association with knowledge and attitude, and it is possible to state that the practice becomes more effective when there is knowledge, evidencing a relationship of dependence between the three domains. In African adolescents, barriers related to misconceptions about the side effects of contraception were observed, mainly the belief that it can cause permanent infertility, in addition to the stigma associated with its use and sexual activity before marriage, as well as the lack of health care focused on the needs for the use of contraceptives ^(^ 2022 ^)^ . 

 In this study, knowledge about the use of the mini-pill as a contraceptive method in the postpartum period for adolescent postpartum women became more consistent because it was mediated by the nurse and through the use of the flipchart. This, being an educational technology, is an essential tool in the education and health process, and the aim is to provide creative teaching that awakens the critical sense of the target audience ^(^ 2022 ^)^ . The use of educational technology favors the improvement of knowledge evidenced in a study carried out with Thai adolescents using educational video for counseling, which demonstrated effectiveness in improving contraceptive knowledge in postpartum adolescents ^(^ 2016 ^)^ . 

 In American adolescents, contraceptive decision-making was found to be influenced by social networks and community, including parents and peers. Mothers played a key role in adolescents’ transition to gaining more autonomy over their reproductive decisions. Providers should consistently present comprehensive contraceptive options to adolescents as a component of preventive health care ^(^ 2022 ^)^ . 

 Participants in a qualitative study conducted in Thailand reported a lack of knowledge about contraception. Although they are aware of the methods, many are not aware of them. Furthermore, despite knowing how to use them, they do not use them, even though they express an intention to do so. In this sense, users need counseling in making decisions to choose the most appropriate contraceptive method and thus avoid unplanned pregnancies ^(^ 2022 ^)^ . 

 For advice, it is worth using the various promotional alternatives for preventing adolescent pregnancy, such as educational technologies that can enhance health promotion, assistance and care, which become useful in the perspective of reaching adolescents in a more assertive way. Here, the flipchart stands out, an educational, attractive and motivating resource that can facilitate the construction of knowledge by adolescent postpartum women about the appropriate use of the contraceptive mini-pill in the postpartum period ^(^ 2020 ^)^ . 

 In this sense, it is up to the nursing professional to intervene in the experience and needs of each adolescent during the postpartum consultation, not limiting themselves to the established protocols ^(^ 2022 ^)^ . 

A limitation of the study is the difficulty in establishing telephone contact with the participants to administer the post-test, although the researcher used the strategy of making three attempts to maintain contact. Also noteworthy is the disadvantage attributed to the quasi-experimental design due to the reduced potential for generalization, with less conclusive results.

The study contributes to nursing in the discussion of the role of the nurse in the sexual orientation of adolescents related to contraceptive methods, contributing to the prevention of unplanned pregnancy or recurrence, so common in adolescence. Therefore, the nurse, inserted in this context, has the role of educator, that is, is important in the implementation of educational practices and contributes to the prevention of STIs, AIDS and unplanned pregnancy, among other needs of the group of adolescents.

## Conclusion

Before the educational intervention, the adolescent postpartum women presented inadequate knowledge, attitudes and practices regarding the use of the mini-pill as a postpartum contraceptive method. After the intervention, they showed significant improvement in the knowledge and practice domains, but the average for the attitude domain considered inadequate increased.

In the association of the domains with the sociodemographic and gynecological-obstetric variables before the intervention, a statistically significant association was detected with the variables age, education, being in school, number of pregnancies and prenatal care. After the intervention, in addition to the variables mentioned, place of residence, knowledge, attitude and practice were also relevant. Age, education, place of residence, number of pregnancies, having had prenatal care and use of the contraceptive method were factors that influenced the adequate use of the mini-pill by the adolescent postpartum women. It is evident that the technology used for educational intervention is effective in promoting knowledge, attitude and practice in preventing and preventing recurrence of pregnancy using the mini-pill as a contraceptive indicated in the postpartum period. However, it needs to be combined with other technologies, as well as with the family and social support network.

Adolescent postpartum women have knowledge, but it is not sufficient for making decisions regarding appropriate practices, probably due to their precocious age or the lack of a family and social support network.
