# Supplementary material for: Knowledge, attitude and practice of puerperal adolescents after an educational intervention on the mini contraceptive pill
Source: Rev Lat Am Enfermagem. 2024 Oct 25;32:e4350. doi: 10.1590/1518-8345.7227.4350 (PMC11526300; doi:10.1590/1518-8345.7227.4350)
Supplement: Translation

# Translation: Conocimiento, actitud y práctica de puérperas adolescentes tras intervención educativa sobre minipíldora anticonceptiva *

**DOI:** 10.1590/1518-8345.7227.4349

**Published:** 2024-10-10

**Authors:** Claudionete Abreu Costa, Ana Karina Bezerra Pinheiro, Lena Maria Barros Fonseca, Tatiane Gomes Guedes, Rita da Graça Carvalhal Frazão Corrêa, Adriana Gomes Nogueira Ferreira

**Affiliations:** ^1^ Universidade Federal do Maranhão, São Luís, MA, Brasil.; ^2^ Universidade Federal do Ceará, Departamento de Enfermagem, Fortaleza, CE, Brasil.; ^3^ Universidade Federal de Pernambuco, Recife, PE, Brasil.

**Keywords:** Adolescente, Periodo Posparto, Anticoncepción Hormonal, Lactancia Materna, Enfermería, Tecnologia Educacional

## Abstract

avaliar la efectividad de una intervención educativa en el conocimiento, la actitud y la práctica de puérperas adolescentes sobre minipíldora como método de contracepción en el posparto.

estudio cuasi experimental con pre y post-tests realizado con 139 puérperas adolescentes en cuatro maternidades públicas, utilizando un instrumento con preguntas sociodemográficas/gineco-obstétrica y una encuesta dirigida llamada Conocimiento, Actitud y Práctica. En el análisis, se utilizaron: *software Statistical Package for the Social Sciences* , versión 20.1 para *Windows* ; nivel de significancia del 5%; intervalo de confianza del 95%; prueba Shapiro-Wilk para probar la normalidad de los datos y test *t* pareado.

los puntajes medios del instrumento Conocimiento, Actitud y Práctica resultaron inadecuados antes de la intervención educativa; después de la intervención, permanecieron inadecuados, pero con discreta mejora; la actitud mostró una inadecuación acentuada. Dicho instrumento, antes de la intervención, se asoció con edad, escolaridad, número de embarazos y haber realizado prenatal; después de la intervención, además de estas, tuvo asociación con otras variables, como lugar de residencia, estar estudiando y haber usado método contraceptivo. Los tres dominios tuvieron asociación entre sí antes y después de la intervención.

la intervención educativa, mediada por la enfermera, fue efectiva para promover conocimiento, actitud y práctica de las puérperas adolescentes en el uso adecuado de la minipíldora como contracepción posparto.

## Destacados

**(1)** Asociación estadística significativa en las variables sociodemografica y gineco-obstétricas.

**(2)** Las puérperas tenían conocimiento, actitudes y prácticas inadecuadas sobre la minipíldora.

**(3)** Después de dos meses, se observó un aumento del conocimiento y la práctica de las puérperas.

**(4)** Los puntajes medios Conocimiento, Actitud y Práctica antes de la intervención y después de 60 días se mantuvieron inadecuados.

**(5)** La tecnología utilizada en la intervención educativa es eficaz para promover los dominios Conocimiento, Actitud y Práctica.

## Introducción

 Brasil presentó, según la Organización Panamericana de la Salud (OPAS), una de las tasas más altas de embarazo adolescente en América Latina en 2016, siendo 68,4 nacidos vivos por cada mil niñas de 15 a 19 años ^(^ 2018 ^)^ . A pesar de que el porcentaje de embarazos en la adolescencia ha disminuido a lo largo de los años en todas las regiones del mundo (aunque de manera desigual), la reincidencia de embarazos permanece estable – alrededor del 20% ^(^ 2020 ^-^ 2020 ^)^ . 

 En 2010, casi el 20% de todos los nacimientos en Brasil eran de parturientas adolescentes, mientras que en 2019 la proporción fue del 14,72%. La mayor disminución se dio en el grupo de edad de 15 a 19 años. Las adolescentes más jóvenes, de 10 a 14 años, presentaron proporciones por debajo del 1% y con tendencia a una disminución más discreta ^(^ 2018 ^)^ . 

 Estudio de geoprocesamiento que analizó la variación espacial del embarazo adolescente en Brasil evidenció que las Regiones Norte, Centro-Oeste y Nordeste presentaron medianas más elevadas de fecundidad adolescente en mujeres de 15 a 19 años ^(^ 2021 ^)^ . En la Región Nordeste, en 2020, Maranhão fue responsable de 23.132 partos de madres adolescentes, y de estos, el municipio de São Luís registró 1.932 nacimientos y São José de Ribamar 498 partos de madres con edad entre 10 a 19 años ^(^ 2023 ^)^ . 

 La falta o la práctica deficiente del planeamiento reproductivo en la adolescencia se ha constituido en un fuerte aliado para el retardo en la disminución de los porcentajes de embarazos en la adolescencia y en la inestabilidad de la reincidencia del embarazo ^(^ 2020 ^)^ , sin embargo las dificultades relacionadas con la adhesión, en lo que se refiere al uso de los métodos anticonceptivos por las madres adolescentes, representan algunos de los factores que mantienen las tasas de embarazo elevadas, especialmente de reincidencia ^(^ 2020 ^,^ 2022 ^-^ 2023 ^)^ . 

 En relación al conocimiento específico sobre los métodos de anticoncepción hormonales, las adolescentes reconocen que la píldora anticonceptiva no previene Infecciones Sexualmente Transmisibles (IST), sin embargo, ellas no saben distinguir la acción de los anticonceptivos orales, incluyendo la minipíldora. Las minipíldoras inhiben el embarazo exclusivamente por medio de efectos progestacionales y son recomendadas durante la lactancia ^(^ 2021 ^-^ 2021 ^)^ . 

 El asesoramiento anticonceptivo es una estrategia importante, y las diversas tecnologías (talleres, folletos educativos, álbum, teléfono) deben ser utilizadas para facilitar el intercambio de información y experiencias, lo que ofrece el conocimiento, para la adhesión al método anticonceptivo consciente ^(^ 2020 ^)^ . El enfermero puede utilizarlas con creatividad en educación y salud, a fin de alcanzar sus objetivos para la adopción de comportamientos saludables, identificando deficiencias y fragilidades y contribuyendo al conocimiento y las prácticas saludables ^(^ 2022 ^)^ . 

 Entre las tecnologías educativas, se destaca el álbum seriado, formado por páginas secuenciales con ilustraciones, mapas, mensajes únicos y directos, utilizado en conferencias, reuniones y en el apoyo de clases. Este recurso ha demostrado ser un excelente medio de divulgar el conocimiento a todos los públicos (adultos jóvenes y adolescentes), siendo muy adecuado para la realización de acciones educativas sin perjudicar la interacción entre el educador y su público ^(^ 2020 ^)^ . 

Es importante, por tanto, la realización de estudios que testeen la efectividad de tecnologías educativas, como el álbum seriado aquí elucidado, y, así, garanticen información segura de modo a repercutir positivamente en el conocimiento, la actitud y la práctica anticonceptiva de puérperas adolescentes.

Por lo expuesto, se objetivó evaluar la efectividad de una intervención educativa en el conocimiento, la actitud y la práctica de puérperas adolescentes sobre minipíldora como método de anticoncepción en el posparto.

## Método

### Diseño del estudio

 Se trata de un estudio evaluativo, del tipo conocimiento, actitud y práctica (CAP) ^(^ 2010 ^)^ , con diseño cuasi-experimental con grupo único, del tipo antes y después ^(^ 2019 ^)^ , de corte transversal ^(^ 2015 ^)^ , utilizando el álbum seriado. 

### Período y lugar

La recolección de datos, realizada en el período de abril a julio de 2022, tuvo lugar en cuatro maternidades públicas, siendo tres en el municipio de São Luís (MA) y una en el municipio de São José de Ribamar (MA). Dos maternidades estaban caracterizadas como de alta complejidad para la atención de gestantes de riesgos y las otras dos para la atención de gestantes de riesgo habitual. Esta opción permitió identificar el CAP de puérperas asistidas en maternidades con perfiles diferentes.

### Población y muestra

Se optó por incluir adolescentes de 10 a 19 años, de acuerdo con la definición de la Organización Mundial de la Salud (OMS), que circunscribe la adolescencia a la segunda década de vida. Dichas adolescentes, independientemente del tipo de embarazo o parto, se encontraban internadas en el alojamiento conjunto. No fueron incluidas puérperas adolescentes que sufrieron cualquier tipo de violencia en el período gestacional puerperal y que se encontraban en área de aislamiento por enfermedad infectocontagiosa. La muestra, compuesta de 139 puérperas adolescentes que respondieron el pre y el post-test después de participar en la intervención educativa, fue obtenida por muestreo no probabilístico consecutivo. El pre-test se realizó con puérperas al menos 12 horas post-parto y el post-test con hasta 6 semanas de puerperio.

El estudio se organizó en cuatro fases:

- Fase I – organización del álbum seriado, con contenido seleccionado a partir del Cuaderno de Atención Básica del Ministerio de Salud sobre salud sexual y reproductiva y de Tarjetas de Asesoramiento sobre Métodos Anticonceptivos para adolescentes, referentes al uso de la minipíldora. El álbum seriado se configura como un material educativo basado en conocimiento científico utilizado como herramienta en actividades de orientación y educación en salud. Tras la adaptación, el álbum fue probado con la participación de un grupo de adolescentes puérperas, que no fueron incluidas en la investigación, con el objetivo de evaluar su aplicación, permitiendo ajustes que favorecieran la mejor comprensión de las participantes.
- Fase II – reclutamiento de las participantes y aplicación del pre-test, utilizando un instrumento con preguntas sociodemográficas (relacionadas a edad, residencia, escolaridad) y gineco-obstétricas (sobre uso de anticonceptivo, número de embarazo, prenatal) y el cuestionario CAP (preguntas sobre conocimiento, actitudes y prácticas relacionadas con la anticoncepción mediante el uso de la minipíldora).
- Fase III – aplicación de la intervención educativa utilizando el álbum seriado y esclarecimiento de dudas sobre las cuestiones del pre-test.
- Fase IV – post-test, realizado utilizando como herramienta el teléfono celular para la aplicación del cuestionario CAP.

El álbum seriado está compuesto de tres hojas, ilustrado con figuras femeninas, siendo una puérpera adolescente dialogando con la enfermera. La primera hoja contiene preguntas y respuestas referentes al conocimiento sobre el uso correcto de la minipíldora para anticoncepción en el posparto, de modo que el intercambio de información se hace de forma lúdica. En la segunda hoja, el diálogo expresa el papel activo de la puérpera en la toma de decisión para el uso correcto de la minipíldora, como forma de recibir el primer *feedback* del conocimiento adquirido. La tercera hoja trata de la práctica de esa puérpera adolescente después de la intervención educativa realizada por la enfermera, al demostrar la comprensión sobre la temática y la práctica del uso adecuado de la minipíldora. En esta hoja del álbum, solo aparece la figura de la puérpera adolescente como protagonista de sus elecciones, es decir, de sus prácticas adecuadas sobre el método. 

### Recolección de datos

Para la recolección de datos, realizada en dos momentos distintos, el pre y el post-test, la investigadora contó con la ayuda de becarios debidamente entrenados. El pre-test se llevó a cabo de manera presencial, durante la estancia en el alojamiento conjunto, de acuerdo con el momento sugerido, de modo que se evitara el período de visitas, para priorizar la individualidad. En el primer contacto, se presentaron la propuesta, la dinámica de la intervención educativa, las etapas de la recolección de datos y la clarificación para mejorar el conocimiento sobre el uso correcto de la minipíldora anticonceptiva, buscando la prevención de la reincidencia de un embarazo no planificado.

 Después de la aplicación del pre-test, se entregaron a las puérperas instrucciones para la etapa subsiguiente relacionada con el post-test, con uso del teléfono celular hasta 6 semanas después del parto ^(^ 2016 ^)^ . 

Inicialmente, la investigadora presentaba el álbum seriado a las puérperas e informaba que allí estaban las mismas cuestiones que ya habían respondido en el pre-test. Las puérperas las observaban atentamente durante 5 minutos, verificando ya las respuestas que habían acertado o errado. Tras las explicaciones, aclaraban sus dudas. Las orientaciones se realizaban en secuencia, del conocimiento a la práctica, con una duración de 20 minutos cuando era individualizada, y 40 minutos cuando era posible un círculo de conversación entre ellas y la investigadora.

En el segundo momento, se aplicó el post-test, incluyendo el CAP. Para conducir esta etapa, se organizó una hoja de cálculo con los datos de cada participante, incluyendo contacto telefónico, fecha del parto y de la aplicación del pre-test. La llamada telefónica a las participantes proseguía con la identificación de la investigadora, de la actividad que se iba a realizar y del recuerdo del pre-test que se realizó en el momento de la internación, seguida de las preguntas del post-test (encuesta CAP). Se hacían hasta tres intentos, cuando la llamada no era contestada en la primera vez. Cada conversación duraba de 10 a 15 minutos. Las respuestas se marcaban en un nuevo instrumento identificado como post-test, con la respectiva fecha, y se registraban en la hoja de cálculo para la organización del banco de datos.

### Instrumento utilizado

 Se utilizó el instrumento de encuesta CAP para evaluar las tres dimensiones (CAP) ^(^ 2019 ^)^ , aplicado en dos momentos: antes y después de la intervención educativa, para medir el resultado principal referente a los tres dominios (CAP) ^(^ 2018 ^-^ 2018 ^)^ de las puérperas adolescentes en cuanto al uso de la minipíldora anticonceptiva. 

El conocimiento se consideraba adecuado si la participante respondía sobre la minipíldora: “la píldora indicada para la mujer que está amamantando es la minipíldora”; “si la mujer presenta vómitos y/o diarrea durante más de 24 horas debe hacer uso del condón hasta el próximo ciclo menstrual”; “algunos medicamentos interfieren en el efecto de la minipíldora”; “si la mujer vomita dentro de 1 hora después de tomar la píldora, es necesario tomar otra píldora”; “la píldora debe ser tomada todos los días, en el mismo horario”; “para comenzar a tomar la píldora, es necesario consultar y tener la prescripción médica”; “cuando una caja de comprimidos termina, la mujer debe iniciar inmediatamente una nueva caja, sin intervalo”; “la minipíldora no debe ser iniciada desde el primer día del posparto”; tomar la minipíldora, la menstruación puede tener alguna alteración”. El instrumento se consideraba adecuado si tenía de siete a nueve correlaciones correctas e inadecuado si tenía menos de siete correlaciones correctas.

La actitud se consideraba adecuada si la puérpera adolescente refería: “tener intención de usar un método anticonceptivo y/o la minipíldora mientras esté amamantando y la píldora posteriormente”; “afirmar que va a consultar un profesional de salud para iniciar el uso de la minipíldora/píldora”; “iniciar el uso de la minipíldora 6 semanas después del parto”. La actitud se consideraba inadecuada si la participante refería no tener intención de usar la minipíldora por cualquier motivo.

La práctica se consideraba adecuada si la puérpera adolescente afirmaba que iba “a usar la minipíldora solo tras consulta médica y con prescripción”; “empezar a tomar la minipíldora después de 6 semanas del posparto, tomando todos los días siempre a la misma hora”; “si olvida tomar una píldora, tomar la olvidada tan pronto como recuerde y la píldora siguiente en el horario habitual”; “cuando termine de tomar una caja de minipíldora, iniciar una nueva sin intervalo”. La práctica se consideraba inadecuada si la adolescente decía que “no va a usar la minipíldora al iniciar las actividades sexuales después del parto, o empezar a tomar por su propia cuenta”.

### Análisis

 Para el análisis de los datos, se aplicaron estadísticas descriptivas con variables dependientes (CAP) acerca de la minipíldora y variables independientes (variables sociodemográficas y obstétricas) para establecer los tipos de actividades realizadas, asociadas a la adopción de comportamiento no deseado. Tras la recolección, los datos fueron codificados y almacenados en una hoja de cálculo de *Excel* , versión 20.1. Inicialmente, se realizaron análisis descriptivos (frecuencia, promedio, desviación estándar, mínimo y máximo) de las variables, los resultados fueron presentados, y el análisis estadístico se llevó a cabo en el *software* *Statistical Package for the Social Sciences* , versión 20.1 para *Windows* , asignándose un nivel de significancia del 5% y un intervalo de confianza del 95%. El test de Shapiro-Wilk se utilizó para probar la normalidad de los datos y el test *t* pareado, para verificar diferencias del CAP antes de la intervención y después de la intervención, considerando un valor de p menor o igual a 0,05. 

### Aspectos éticos

 Los aspectos éticos referentes a la investigación con seres humanos fueron todos respetados como determina la resolución 466/12 del Ministerio de Salud y sus complementarias ^(^ 2013 ^)^ . La investigación fue aprobada por el Comité de Ética e Investigación del Hospital Universitario de la Universidad Federal de Maranhão (protocolo 4.988.517). 

## Resultados

Durante la recolección, 151 puérperas adolescentes realizaron el pre-test; sin embargo, en la etapa de aplicación de los post-tests, hubo una pérdida de 12 participantes, sumando un total de 139 puérperas adolescentes en la muestra.

 Los datos antes de la intervención mostraron que el conocimiento, la actitud y la práctica fueron considerados adecuados, con 33,81%, 15,82% y 19,42%, respectivamente, con un puntaje medio entre los tres dominios de 32 para “adecuado” ( Tabla 1 ). 

**Tabla 1:** - Conocimiento, actitud y práctica de las puérperas adolescentes antes de la intervención educativa. São Luís y São José de Ribamar, MA, Brasil, 2023

CAP*	**n**	**DE** ^†^	**Mínima**	**Máxima**	**Puntaje**		**Total**
					**Adecuado** **n (%)**	**Inadecuado** **n (%)**	
Conocimiento	139	2,68	-	29	47 (33,81)	92 (66,18)	139
Actitud	139	3,14	01	28	22 (15,82)	117 (84,17)	139
Práctica	139	4,97	-	31	27 (19,42)	112 (80,57)	139
Promedio CAP*					32	107	139

* CAP = Conocimiento, Actitud y Práctica

^†^ DE = Desviación Estándar

 Tras la intervención, se observó un aumento entre los tres dominios CAP. Hubo una disminución en el promedio de adecuado a 51, sin embargo, cuando se analizó aisladamente el dominio de actitud, se observó un aumento en el número de participantes con actitudes inadecuadas ( Tabla 2 ). 

 En la asociación del CAP con las variables sociodemográficas y gineco-obstétricas antes de la intervención hubo una asociación estadísticamente significativa para las variables edad, escolaridad, estar estudiando, número de embarazo y realización de prenatal (p=0,05) ( Tabla 3 ). 

En la asociación de los CAP adecuados con las variables sociodemográficas y gineco-obstétricas después de la intervención, hubo una asociación estadísticamente significativa entre las variables edad, escolaridad, número de embarazos, realizó prenatal, lugar de residencia y estar estudiando. En cuanto a la variable usó algún método anticonceptivo, solo hubo asociación con el dominio actitud para las variables edad, escolaridad, número de embarazos, realizó prenatal, lugar de residencia y estar estudiando.

 En la asociación entre los dominios conocimiento, actitud y práctica antes de la intervención, hubo una asociación estadísticamente significativa entre la actitud y la práctica (p<0,05). Después de la intervención, hubo una asociación significativa entre los tres dominios: la práctica con el conocimiento (p<0,05) y la actitud (p=0,05) ( Tabla 5 ). 

**Tabla 2:** - Conocimiento, actitud y práctica de las puérperas adolescentes después de la intervención educativa. São Luís y São José de Ribamar, MA, Brasil, 2023

CAP*	**n**	**DE** ^†^	**Mínima**	**Máxima**	**Puntaje**		**Total**
					**Adecuado** **n (%)**	**Inadecuado** **n (%)**	
Conocimiento	139	2,59	-	37	90 (64,74)	49 (35,25)	139
Actitud	139	3,18	-	35	13 (9,35)	126 (90,64)	139
Práctica	139	3,26	-	36	51 (36,69)	88 (63,30)	139
Promedio CAP					51	88	139

* CAP = Conocimiento, Actitud y Práctica

^†^ DE = Desviación Estándar

**Tabla 3:** - Asociación del conocimiento, de la actitud y de la práctica con variables sociodemográficas y gineco-obstétricas de puérperas adolescentes antes de la intervención educativa, por análisis de correlación. São Luís y São José de Ribamar, MA, Brasil, 2023

**Variables**	**Total**	**Conocimiento adecuado**		**Actitud adecuada**		**Práctica adecuada**	
		**n (%)**	**Valor de p**	**n (%)**	**Valor de p**	**n (%)**	**Valor de p**
**Edad (años)**							
12-13	28	2 (7,1)		2 (7,1)		2 (7,1)	
14-15	51	1 (1,9)		1 (1,9)		2 (3,9)	
16-17	52	6 (11,5)		-		4 (7,6)	
18-19	8	6 (75,0)		6 (75,0)		5 (62,5)	
**Residencia, zona**							
Rural	61	5 (8,19)	0,31	4 (6,5)	0,23	5 (8,1)	0,11
Urbana	78	8 (10,25)		7 (10,2)		9 (11,5)	
**Escolaridad**							
Educación Primaria completa	2	1 (50,0)	0,05	-	0,05	-	0,05
Educación Primaria incompleta	4	3 (75,0)		2 (50,0)		-	
Educación Primaria Superior completa	2	-		-		-	
Educación Primaria Superior incompleta	58	15 (25,8)		10 (17,2)		8 (13,7)	
Secundaria completa	31	13 (41,9)		7 (22,5)		5 (16,1)	
Secundaria incompleta	42	12 (28,5)		9 (21,4)		6 (14,2)	
**Estudiando actualmente**							
Sí	39	12 (30,8)	0,01	9 (23,1)	0,18	9 (23,1)	0,14
No	100	5 (5,0)		4 (4,0)		7 (7,0)	
**Método anticonceptivo**							
Sí	14	5 (35,7)	0,33	6 (42,9)	0,05	6 (42,9)	0,33
No	125	12 (9,6)		11 (8,8)		21 (16,8)	
**Embarazo**							
1	98	18 (18,4)	0,05	21 (21,4)	0,05	15 (15,3)	0,05
2	27	13 (48,1)		15 (55,6)		12 (44,4)	
3 o más	14	3 (42,2)		3 (33,3)		1 (11,1)	
**Realizó prenatal**							
Sí	116	29 (25,0)	0,05	20 (17,2)	0,05	22 (19,0)	0,05
No	23	8 (34,7)		5 (21,7)		7 (30,4)	

**Tabla 4:** - Asociación del conocimiento, de la actitud y de la práctica con variables sociodemográficas y gineco-obstétricas de puérperas adolescentes después de la intervención educativa, por análisis de correlación. São Luís y São José de Ribamar, MA, Brasil, 2023

**Variables**	**Total**	**Conocimiento adecuado**		**Actitud adecuada**		**Práctica adecuada**	
		**n (%)**	**Valor de p**	**n (%)**	**Valor de p**	**n (%)**	**Valor de p**
**Edad (años)**							
12-13	28	4 (14,3)		4 (14,3)		4 (14,3)	
14-15	51	3 (5,8)		3 (5,8)		4 (7,8)	
16-17	52	10 (19,2)		2 (3,8)		6 (11,5)	
18-19	8	3 (37,5)		2 (25,0)		1 (12,5)	
**Residencia, zona**							
Rural	61	7 (11,5)	0,05	6 (9,8)	0,01	7 (11,5)	0,01
Urbana 2	78	10 (12,8)		9 (11,5)		11 (14,1)	
**Escolaridad**							
Educación Primaria completa	2	-	0,05	-	0,05	-	0,05
Educación Primaria incompleta	4	2 (50,0)		2 (50,0)		2 (50,0)	
Educación Primaria Superior completa	2	-		-		-	
Educación Primaria Superior incompleta	58	17 (29,3)		12 (20,7)		10 (17,2)	
Secundaria completa	31	15 (48,4)		9 (29,0)		7 (22,6)	
Secundaria incompleta	42	14 (33,3)		11 (26,2)		8 (19,0)	
**Estudiando actualmente**							
Sí	39	14 (35,9)	0,04	11 (28,2)	0,04	9 (23,1)	0,05
No	100	7 (7,0)		6 (6,0)		7 (7,0)	
**Método anticonceptivo**							
Sí	14	6 (42,9)	0,31	7 (50,0)	0,04	7 (50,0)	0,17
No	125	13 (10,4)		12 (9,6)		22 (17,6)	
**Embarazo**							
1	98	20 (20,4)	0,05	23 (23,5)	0,05	17 (17,3)	0,05
2	27	15 (55,6)		17 (63,0)		14 (51,9)	
3 o más	14	7 (50,0)		7 (50,0)		5 (35,7)	
**Realizó prenatal**							
Sí	116	31 (26,7)	0,05	35 (30,1)	0,05	24 (20,7)	0,05
No	23	10 (43,4)		7 (30,4)		9 (39,1)	

**Tabla 5:** - Asociación entre los dominios conocimiento, actitud y práctica de puérperas adolescentes antes y después de la intervención educativa. São Luís y São José de Ribamar, MA, Brasil, 2023

	**Adecuado** **n (%)**	**Inadecuado** **n (%)**	**Valor de p**
**Práctica antes**			
**Conocimiento**			
Adecuado	1 (0,71)	11 (7,91)	0,12
Inadecuado	3 (2,15)	26 (19,07)	
**Actitud**			
Adecuado	1 (0,71)	15 (10,79)	0,04
Inadecuado	2 (1,43)	36 (25,89)	
**Práctica después**			
**Conocimiento**			
Adecuado	21 (15,10)	14 (10,07)	0,04
Inadecuado	15 (10,79)	29 (20,86)	
**Actitud**			
Adecuado	1 (0,71)	10 (7,19)	0,05
Inadecuado	5 (3,59)	17 (12,23)	

## Discusión

 El puerperio es un momento del ciclo gravídico-puerperal oportuno para prevenir, identificar y tratar alteraciones que comprometen la salud de la mujer ^(^ 2022 ^)^ , como por ejemplo la reincidencia del embarazo no planificado en la adolescencia. 

 El presente estudio mostró que la distribución de los puntajes relacionados con las medias de los dominios conocimiento, actitudes y práctica respecto al uso de la minipíldora antes de la intervención educativa fueron bajos, evidenciando conocimiento inadecuado en relación a los métodos anticonceptivos y a la vulnerabilidad de las puérperas adolescentes, entre ellos la minipíldora como método de prevención de reincidencia de embarazo en el posparto. Un estudio cualitativo realizado en Tailandia con adolescentes sobre la reincidencia de gestación identificó que las motivaciones fueron la falta de anticonceptivo, de conocimiento sobre métodos y de conciencia, que se caracterizó por saber cómo usar, pero no usar y por pretender usar y no usar ^(^ 2023 ^)^ . 

 Un estudio nacional realizado en Pernambuco también destaca el preservativo masculino como el más conocido, seguido de los anticonceptivos orales, pero las participantes no sabían qué tipo de píldora utilizar, cuándo y cómo utilizarlas ^(^ 2022 ^)^ , coincidiendo con el presente estudio y demostrando fragilidad en la actitud de estos adolescentes en poner sus conocimientos en práctica debido a la falta de información. 

 El dominio actitud de la puérpera adolescente, después de la intervención educativa, aumentó el puntaje medio de inadecuación en relación a los otros dominios que se mantuvieron inadecuados, pero presentando mejoras. Se cree que la intervención realizada elevó el conocimiento de las participantes sobre el uso adecuado de la minipíldora, pero no fue suficiente para el cambio de actitud hacia una práctica adecuada. Es importante complementar el cuidado con intervenciones educativas de modo a mejorar el conocimiento, conforme observado en un estudio que utilizó video educativo en Tailandia, como complemento al método tradicional de asesoramiento en consultorio, lo cual aumentó significativamente el conocimiento sobre el método anticonceptivo y, consecuentemente, contribuyó a la preferencia por los métodos presentados ^(^ 2022 ^)^ . 

 Al analizar la asociación entre las variables sociodemográficas con el conocimiento, la actitud y la práctica adecuados de las puérperas antes de la intervención educativa, se verificó que las variables edad y escolaridad presentaron asociación significativa en los tres dominios, así como la variable estar estudiando, en el dominio conocimiento, demostrando que la mayor franja etaria y estar estudiando son indicadores que favorecen la capacidad para comprender y mejorar el conocimiento, la actitud y la práctica adecuada de las participantes. Después de la intervención, se percibió que, además de la edad y la escolaridad, otras variables estudiadas, como el lugar de residencia, tuvieron asociaciones significativas en los tres dominios. En este contexto, ciertamente, el uso del álbum seriado en forma impresa, mediado por la enfermera en la relación interaccional con las participantes, fue muy importante, configurando una tecnología educativa que sirve para facilitar la adquisición del CAP de las puérperas adolescentes sobre el uso del método anticonceptivo en cuestión ^(^ 2020 ^)^ . 

 Una investigación enfocada en el conocimiento, actitudes y prácticas contraceptivas entre adolescentes reveló lagunas que requieren atención para prevenir el embarazo no planificado en la adolescencia, que ocurre con frecuencia en el posparto y puede suceder en el puerperio tardío (del 11º al 45º día) ^(^ 2023 ^)^ . Se resalta la importancia de la orientación de los padres sobre la contracepción antes del inicio de la vida sexual, así como el papel de los profesionales de salud, para discutir, de forma proactiva, la contracepción mediante la comunicación individualizada y confidencial ^(^ 2023 ^)^ . 

 Estudios nacionales e internacionales han evidenciado la asociación de la reincidencia del embarazo en la adolescencia con características socioeconómicas y demográficas de la población; mujeres solteras; baja renta; escolaridad inadecuada para la edad y dificultad de acceso a las acciones de planificación reproductiva, especialmente en el período posparto, reiterando las desigualdades sociales existentes entre la población. En este contexto, se destaca el acceso desigual de la población femenina a la planificación reproductiva ^(^ 2022 ^)^ . Se subraya que el embarazo en la adolescencia está asociado al aumento de complicaciones maternas y fetales y también es responsable del aumento de la incidencia de abortos inseguros y de la mortalidad materna. Así, es importante concienciar sobre los métodos efectivos de contracepción posparto para prevenir el embarazo en esta fase de la vida ^(^ 2022 ^)^ . 

En el análisis de la asociación entre las variables gineco-obstétricas con el conocimiento, la actitud y la práctica adecuados de las puérperas, antes de la intervención educativa, se verificó que las variables número de embarazos y haber realizado prenatal presentaron asociación significativa en los tres dominios. Después de la intervención, además de las dos variables mencionadas, el uso de método anticonceptivo tuvo asociación significativa en el dominio actitud. Este resultado muestra que la intervención educativa fue fructífera, en el sentido de posibilitar la comprensión sobre el método, y favoreció la adhesión a la práctica del uso adecuado de la minipíldora.

 Resultados de un estudio realizado con adolescentes y mujeres jóvenes en Guinea revelaron factores individuales y comunitarios asociados a la necesidad no atendida de contracepción. Los autores sugieren que, además de las campañas de educación sexual y concienciación a través de los medios y los lugares donde las mujeres se reúnen, el asesoramiento individual en servicios de salud debe ser mejor dirigido a este público, considerando sus características individuales y contextuales ^(^ 2023 ^)^ . 

 Estos indicadores, asociados con los factores socioeconómicos, probablemente influyeron positivamente en despertar la atención de la puérpera para mejorar su conocimiento, principalmente su actitud hacia una práctica adecuada del uso de la minipíldora como método anticonceptivo en el posparto ^(^ 2019 ^)^ . 

 Al analizar la asociación entre los tres dominios, se verificó una asociación significativa en la relación de la actitud con la práctica adecuada, siendo posible inferir que la toma de decisiones de la participante para la práctica del método presentado tiene relación con los conocimientos adquiridos previamente y se da por sus propias necesidades de usar algún método preventivo. Después de la intervención educativa, la práctica tuvo asociación significativa con el conocimiento y con la actitud, siendo posible afirmar que la práctica se vuelve más efectiva cuando hay conocimiento, evidenciando una relación de dependencia entre los tres dominios. En adolescentes africanos, se observaron barreras relacionadas con las concepciones equivocadas sobre los efectos secundarios de la contracepción, principalmente la creencia de que esta puede causar infertilidad permanente, además del estigma asociado a su uso y a la actividad sexual antes del matrimonio, así como la falta de asistencia sanitaria orientada a las necesidades para el uso de anticonceptivos ^(^ 2022 ^)^ . 

 En este estudio, el conocimiento del uso de la minipíldora como método anticonceptivo en el posparto para la puérpera adolescente se volvió más consistente por haber sido mediado por la enfermera y por el uso del álbum seriado. Este, siendo una tecnología educativa, es una herramienta esencial en el proceso de educación y salud, si se aspira a una enseñanza creativa que despierte el sentido crítico del público objetivo ^(^ 2022 ^)^ . El uso de tecnología educativa favorece la mejora del conocimiento evidenciado en un estudio realizado con adolescentes tailandeses utilizando video educativo para asesoramiento, el cual demostró efectividad para mejorar el conocimiento anticonceptivo en adolescentes posparto ^(^ 2016 ^)^ . 

 En adolescentes norteamericanas, se identificó que la toma de decisiones anticonceptivas fue influenciada por las redes sociales y la comunidad, incluyendo los padres y los amigos. Las madres desempeñaron un papel fundamental en la transición de los adolescentes para ganar más autonomía sobre sus decisiones reproductivas. Los proveedores deben presentar consistentemente a los adolescentes opciones anticonceptivas comprensivas como un componente de los cuidados de salud preventivos ^(^ 2022 ^)^ . 

 Participantes del estudio cualitativo realizado en Tailandia indicaron falta de conocimiento sobre anticoncepción. Aunque conocen los métodos, muchos no tienen conciencia sobre ellos. Además, a pesar de saber usarlos, no los utilizan, incluso expresando la intención de hacerlo. En este sentido, las usuarias necesitan asesoramiento en la toma de decisiones para elegir el método anticonceptivo más adecuado y así evitar embarazos no planificados ^(^ 2022 ^)^ . 

 Para el asesoramiento, vale recurrir a diversas alternativas promocionales de la prevención de embarazo en la adolescencia, como las tecnologías educativas que son capaces de potenciar la promoción, la asistencia y el cuidado con la salud, que se vuelven útiles desde la perspectiva de alcanzar a las adolescentes de forma más asertiva. Aquí, se destaca el álbum seriado, recurso didáctico, atractivo y motivador que puede facilitar la construcción del conocimiento por las puérperas adolescentes sobre el uso adecuado de la minipíldora anticonceptiva en el posparto ^(^ 2020 ^)^ . 

 En este sentido, corresponde al profesional de enfermería intervenir en la vivencia y en las necesidades de cada adolescente durante la consulta puerperal, no limitándose solo a los protocolos establecidos ^(^ 2022 ^)^ . 

Se destaca como limitación del estudio la dificultad en establecer contacto telefónico con las participantes para la aplicación del post-test, aunque la investigadora utilizó la estrategia de realizar tres intentos para mantener contacto. Aún se destaca la desventaja atribuida al diseño cuasi experimental por el potencial de generalización reducido, con resultados menos concluyentes.

El estudio aporta una contribución a la enfermería en la discusión del papel del enfermero en la orientación sexual del adolescente relacionada con los métodos anticonceptivos, contribuyendo a la prevención del embarazo no planificado o la ocurrencia de reincidencia, tan común en el período de la adolescencia. Por lo tanto, el enfermero, insertado en este contexto, tiene un papel de educador, es decir, es importante en la actuación de las prácticas educativas y contribuye a la prevención de ITS, VIH, embarazo no planeado, entre otras necesidades del grupo de adolescentes.

## Conclusión

Las puérperas adolescentes presentaron, antes de la intervención educativa, conocimientos, actitudes y prácticas inadecuados respecto al uso de la minipíldora como método de anticoncepción posparto. Después de la intervención, mostraron una mejora significativa en los dominios conocimiento y práctica, sin embargo, el promedio del dominio actitud considerado inadecuado aumentó.

En la asociación de los dominios con las variables sociodemográficas y gineco-obstétricas antes de la intervención, se detectó una asociación estadísticamente significativa con las variables edad, escolaridad, estar estudiando, número de embarazos y prenatal. Después de la intervención, además de las variables mencionadas, el lugar de residencia, el conocimiento, la actitud y la práctica también presentaron relevancia. La edad, la escolaridad, el lugar de residencia, el número de embarazos, haber realizado prenatal y el uso del método anticonceptivo fueron factores que influyeron en el uso adecuado de la minipíldora por las puérperas adolescentes.

Se evidencia que la tecnología empleada para la intervención educativa es efectiva para la promoción del conocimiento, la actitud y la práctica en la prevención y la reincidencia del embarazo utilizando la minipíldora como anticonceptivo indicado en el posparto. Sin embargo, es necesaria su asociación con otras tecnologías, así como con la red de apoyo familiar y social.

Las puérperas adolescentes tienen conocimiento, pero este no es suficiente para la toma de decisión de una práctica adecuada, probablemente por la precocidad de la edad o por la falta de la red de apoyo familiar y social.

# Translation: Conhecimento, atitude e prática de puérperas adolescentes após intervenção educativa sobre minipílula anticoncepcional *

**DOI:** 10.1590/1518-8345.7227.4351

**Published:** 2024-10-10

**Authors:** Claudionete Abreu Costa, Ana Karina Bezerra Pinheiro, Lena Maria Barros Fonseca, Tatiane Gomes Guedes, Rita da Graça Carvalhal Frazão Corrêa, Adriana Gomes Nogueira Ferreira

**Affiliations:** ^1^ Universidade Federal do Maranhão, São Luís, MA, Brasil.; ^2^ Universidade Federal do Ceará, Departamento de Enfermagem, Fortaleza, CE, Brasil.; ^3^ Universidade Federal de Pernambuco, Recife, PE, Brasil.

**Keywords:** Adolescente, Período Pós-Parto, Contracepção Hormonal, Aleitamento Materno, Enfermagem, Tecnologia Educacional

## Abstract

avaliar a efetividade de uma intervenção educativa no conhecimento, na atitude e na prática de puérperas adolescentes sobre minipílula como método de contracepção no pós-parto.

estudo quase experimental com pré e pós-testes realizado com 139 puérperas adolescentes em quatro maternidades públicas, utilizando-se um instrumento com questões sociodemográficas/gineco-obstétrica e um inquérito direcionado chamado Conhecimento, Atitude e Prática. Na análise, foram usados: *software* *Statistical Package for the Social Sciences* , versão 20.1 para *Windows* ; nível de significância de 5%; intervalo de confiança de 95%; teste Shapiro-Wilk para testar normalidade dos dados e teste *t* pareado.

os escores médios do instrumento Conhecimento, Atitude e Prática mostraram-se inadequados antes da intervenção educativa; após a intervenção, permaneceram inadequados, mas com discreta melhora; a atitude mostrou-se com inadequação acentuada. Tal instrumento, antes da intervenção, associou-se com idade, escolaridade, número de gravidezes e ter feito pré-natal; após a intervenção, além destas, teve associação com outras variáveis, como local de residência, estar estudando e ter usado método contraceptivo. Os três domínios tiveram associação entre si antes e após intervenção.

a intervenção educativa, mediada pela enfermeira, foi efetiva para promoção de conhecimento, atitude e prática das puérperas adolescentes no uso adequado da minipílula como contracepção pós-parto.

## Destaques

**(1)** Associação estatística significativa nas variáveis sociodemográfica e gineco-obstétricas.

**(2)** As puérperas tinham conhecimento, atitudes e práticas inadequadas sobre a minipílula.

**(3)** Após dois meses, observou-se aumento do conhecimento e prática das puérperas.

**(4)** Os escores médios Conhecimento, Atitude e Prática antes da intervenção e após 60 dias se mantiveram inadequados.

**(5)** A tecnologia usada à intervenção educativa é eficaz na promoção dos domínios Conhecimento, Atitude e Prática.

## Introdução

 O Brasil apresentou, segundo a Organização Pan-Americana de Saúde (OPAS), uma das maiores taxas de gravidez na adolescência da América Latina em 2016, sendo 68,4 nascidos vivos a cada mil meninas de 15 a 19 anos ^(^ 2018 ^)^ . Apesar de o percentual de gestações na adolescência ter diminuído ao longo dos anos em todas as regiões do mundo (ainda que de maneira desigual), a reincidência de gestações permanece estável – em torno de 20% ^(^ 2020 ^-^ 2020 ^)^ . 

 Em 2010, quase 20% de todos os nascimentos no Brasil eram de parturientes adolescentes, enquanto em 2019 a proporção foi de 14,72%. A maior diminuição se deu na faixa etária de 15 a 19 anos. As adolescentes mais jovens, de 10 a 14 anos, apresentaram proporções abaixo de 1% e com tendência à diminuição mais discreta ^(^ 2018 ^)^ . 

 Um estudo de geoprocessamento que analisou a variação espacial da gravidez na adolescência no Brasil evidenciou que as Regiões Norte, Centro-Oeste e Nordeste apresentaram medianas mais elevadas de fecundidade na adolescência em mulheres de 15 a 19 anos ^(^ 2021 ^)^ . Na Região Nordeste, em 2020, o Maranhão foi responsável por 23.132 partos de mães adolescentes; destes, o município de São Luís registrou 1.932 nascimentos e São José de Ribamar 498 partos de mães com idade entre 10 a 19 anos ^(^ 2023 ^)^ . 

 A falta ou a prática deficiente do planejamento reprodutivo na adolescência tem se constituído como uma forte aliada para o retardo na diminuição dos percentuais de gestações na adolescência e na instabilidade da reincidência de gravidez ^(^ 2020 ^)^ , entretanto, as dificuldades relacionadas à adesão, no que se refere ao uso dos métodos contraceptivos pelas mães adolescentes, representam alguns dos fatores que mantêm as taxas de gravidez elevadas, especialmente no que tange à reincidência ^(^ 2020 ^,^ 2022 ^-^ 2023 ^)^ . 

 Em relação ao conhecimento específico sobre os métodos de contracepção hormonais, as adolescentes reconhecem que a pílula anticoncepcional não previne Infecções Sexualmente Transmissíveis (IST), porém elas não sabem distinguir a ação dos anticoncepcionais orais, incluindo a minipílula. As minipílulas inibem a gestação exclusivamente por meio de efeitos progestacionais e são recomendadas durante a amamentação ^(^ 2021 ^-^ 2021 ^)^ . 

 O aconselhamento contraceptivo é uma estratégia importante, e as diversas tecnologias (oficinas, cartilhas educativas, álbum, telefone) devem ser utilizadas para facilitar o compartilhamento de troca de informações e experiências, oportunizando o conhecimento, para a adesão ao método contraceptivo consciente ^(^ 2020 ^)^ . O enfermeiro pode utilizá-las com criatividade em educação e saúde, a fim de alcançar seus objetivos para adoção de comportamentos saudáveis, identificando deficiências e fragilidades e contribuindo para o conhecimento e as práticas saudáveis ^(^ 2022 ^)^ . 

 Dentre as tecnologias educacionais, destaca-se o álbum seriado, formado por páginas sequenciais com ilustrações, mapas, mensagens únicas e diretas, utilizado em palestras, reuniões e no auxílio de aulas. Tal recurso tem se mostrado um excelente meio de divulgar o conhecimento a todos os públicos (adultos jovens e adolescentes), sendo muito adequado para a realização de ações educativas, sem prejudicar a interação entre o educador e seu público ^(^ 2020 ^)^ . 

É importante, pois, a realização de estudos que testem a efetividade de tecnologias educativas, como o álbum seriado aqui elucidado, e, assim, garantir informações seguras, de modo a repercutir positivamente no conhecimento, na atitude e na prática contraceptiva de puérperas adolescentes.

Dado o cenário exposto, objetivou-se, neste estudo, avaliar a efetividade de uma intervenção educativa no conhecimento, na atitude e na prática de puérperas adolescentes sobre minipílula como método de contracepção no pós-parto.

## Método

### Desenho do estudo

 Trata-se de um estudo avaliativo, do tipo conhecimento, atitude e prática (CAP) ^(^ 2010 ^)^ , com desenho quase-experimental com grupo único, do tipo antes e depois ^(^ 2019 ^)^ , de corte transversal ^(^ 2015 ^)^ , com utilização do álbum seriado. 

### Período e local

A coleta de dados foi realizada no período de abril a julho de 2022, em quatro maternidades públicas, sendo três no munícipio de São Luís (MA) e uma no município de São José de Ribamar (MA). Duas maternidades eram caracterizadas como alta complexidade para atendimento de gestante de riscos e as outras duas para atendimento de gestante de risco habitual. Essa opção permitiu identificar o CAP de puérperas assistidas em maternidade com perfis diferentes.

### População e amostra

Optou-se por incluir adolescentes de 10 a 19 anos, conforme a definição da Organização Mundial da Saúde (OMS), que circunscreve a adolescência à segunda década de vida. Tais adolescentes, independentemente do tipo de gravidez ou parto, encontravam-se internadas no alojamento conjunto. Não foram incluídas puérperas adolescentes que sofreram quaisquer tipos de violência no período gravídico puerperal e/ou que se encontravam em área de isolamento por doença infectocontagiosa. A amostra, composta por 139 puérperas adolescentes que responderam o pré e o pós-teste após participação da intervenção educativa, foi obtida por amostragem não probabilística consecutiva. O pré-teste foi realizado com puérperas que passaram por no mínimo 12 horas pós-parto e o pós-teste com até 6 semanas de puerpério.

O estudo foi organizado em quatro fases:

- Fase I – organização do álbum seriado, com conteúdo selecionado a partir do Caderno de Atenção Básica do Ministério da Saúde sobre saúde sexual e reprodutiva e de Cartões de Aconselhamento sobre Métodos Contraceptivos para adolescentes, referentes ao uso da minipílula. O álbum seriado configura-se como um material educativo embasado em conhecimento científico utilizado como ferramenta em atividades de orientação e educação em saúde. Após a adaptação, o álbum foi testado com a participação de um grupo de adolescentes puérperas, as quais não foram incluídas na pesquisa, com o objetivo de avaliar sua aplicação, possibilitando ajuste que favorecesse à melhor compreensão das participantes.
- Fase II – recrutamento das participantes e aplicação do pré-teste, utilizando-se instrumento com questões sociodemográficas (relacionadas a idade, residência, escolaridade) e gineco-obstétricas (sobre uso de contraceptivo, número de gravidez, pré-natal), além do inquérito CAP (questões sobre conhecimento, atitudes e práticas relacionadas à contracepção por meio do uso da minipílula).
- Fase III – aplicação da intervenção educativa utilizando o álbum seriado e esclarecimento de dúvidas sobre as questões do pré-teste.
- Fase IV – pós-teste, realizado utilizando-se como ferramenta o telefone celular para a aplicação do inquérito CAP.

O álbum seriado é composto por três folhas, ilustrado com figuras femininas, como, por exemplo, uma puérpera adolescente dialogando com a enfermeira. A primeira folha contém perguntas e respostas referentes ao conhecimento sobre o uso correto da minipílula para contracepção no pós-parto, de modo que a troca de informação é feita de forma lúdica. Na segunda folha, o diálogo expressa o papel ativo da puérpera na tomada de decisão para o uso correto da minipílula, como forma de receber o primeiro *feedback* do conhecimento adquirido. A terceira folha trata da prática dessa puérpera adolescente após a intervenção educativa realizada pela enfermeira, ao demonstrar a compreensão sobre a temática e a prática do uso adequado da minipílula. Nessa folha do álbum, só aparece a figura da puérpera adolescente como protagonista de suas escolhas, ou seja, de suas práticas adequadas sobre o método. 

### Coleta de dados

Para a coleta de dados, realizada em dois momentos distintos, o pré e o pós-teste, a pesquisadora contou com auxílio de bolsistas devidamente treinados. O pré-teste ocorreu de forma presencial, durante a internação no alojamento conjunto, de acordo como o momento sugerido, de modo que fosse evitado o período de visitas, para priorizar a individualidade. No primeiro contato, apresentou-se a proposta, a dinâmica da intervenção educativa, as etapas da coleta de dados e o esclarecimento para melhorar o conhecimento sobre o uso correto da minipílula anticoncepcional, visando à prevenção da reincidência de uma gravidez não planejada.

 Após a aplicação do pré-teste, as instruções para a etapa subsequente relacionada ao pós-teste foram entregues às puérperas, com utilização do telefone celular até 6 semanas após o parto ^(^ 2016 ^)^ . 

Inicialmente, a pesquisadora apresentava o álbum seriado para as puérperas e informava que ali estavam as mesmas questões que elas já tinham respondido no pré-teste. As puérperas as observavam atentamente durante 5 minutos, conferindo já as respostas que tinham acertado ou errado. Após as explicações, tiravam suas dúvidas. As orientações eram realizadas na sequência, do conhecimento à prática, com duração de 20 minutos, quando realizada de modo individualizado, e 40 minutos, quando era possível a roda de conversa entre elas e a pesquisadora.

No segundo momento, aplicou-se o pós-teste, incluindo o CAP. Para conduzir essa etapa, organizou-se uma planilha com os dados de cada participante, incluindo contato telefônico, data do parto e da aplicação do pré-teste. A chamada telefônica para as participantes seguia com a identificação da pesquisadora, da atividade que iria ser realizada e da lembrança do pré-teste que foi realizado no momento da internação, seguida das perguntas do pós-teste (inquérito CAP). Foram feitas até três tentativas, quando a ligação não era atendida na primeira vez. Cada conversa durava de 10 a 15 minutos. As respostas eram marcadas em um novo instrumento identificado como pós-teste, com a respectiva data, sendo registradas em planilha para a organização do banco de dados.

### Instrumento utilizado

 Utilizou-se o instrumento de inquérito CAP visando à avaliação das três dimensões (CAP) ^(^ 2019 ^)^ , aplicado em dois momentos: antes e após a intervenção educativa, para mensurar o desfecho principal referente aos três domínios (CAP) ^(^ 2018 ^-^ 2018 ^)^ das puérperas adolescentes quanto ao uso da minipílula anticoncepcional. 

O conhecimento era considerado adequado se a participante respondesse sobre a minipílula: “a pílula indicada para a mulher que está amamentando é a minipílula”; “se a mulher apresentar vômitos e/ou diarreia durante mais de 24 horas, deve fazer uso da camisinha até o próximo ciclo menstrual”; “alguns medicamentos interferem no efeito da minipílula”; “se a mulher vomitar dentro de 1 hora após tomar a pílula, é preciso tomar outra pílula”; “a pílula deve ser tomada todos os dias, no mesmo horário”; “para começar a tomar a pílula, é preciso consultar e ter a prescrição médica”; “quando uma cartela de comprimidos termina, a mulher deve iniciar logo uma nova cartela, sem intervalo”; “a minipílula não deve ser iniciada desde o primeiro dia de pós-parto”; “tomando a minipílula, a menstruação pode ter alguma alteração”. O instrumento foi considerado adequado se tivesse sete a nove correlações corretas e inadequado se tivesse menos de sete correlações corretas.

A atitude foi considerada adequada se a puérpera adolescente referisse: “ter intenção de usar um método contraceptivo e/ou a minipílula enquanto estiver amamentando e a pílula posteriormente”; “afirmar que vai consultar um profissional de saúde para iniciar o uso da minipílula/pílula”; “iniciar o uso da minipílula 6 semanas após o parto”. A atitude foi considerada inadequada se a participante referisse não ter intenção de usar a minipílula por qualquer motivo.

A prática foi considerada adequada se a puérpera adolescente afirmasse que ia “usar a minipílula só após consulta médica e com prescrição”; “iniciar a tomar a minipílula após 6 semanas de pós-parto, tomando todos os dias sempre no mesmo horário”; “se esquecer de tomar uma pílula, tomar a esquecida logo que lembrar e a pílula seguinte no horário de costume”; “quando terminar de tomar uma cartela de minipílula, iniciar uma nova sem intervalo”. A prática foi considerada inadequada se a adolescente falasse que “não vai usar a minipílula ao iniciar as atividades sexuais após o parto, ou começar a tomar por contra própria”.

### Análise

 Para a análise dos dados, aplicou-se estatísticas descritivas com variáveis dependentes (CAP) acerca da minipílula e variáveis independentes (variáveis sociodemográficas e obstétricas) para estabelecer os tipos de atividades realizadas, associadas à adoção de comportamento indesejado. Após a coleta, os dados foram codificados e armazenados em planilha do *Excel* , versão 20.1. Inicialmente, realizou-se as análises descritivas (frequência, média, desvio-padrão, mínimo e máximo) das variáveis, os resultados foram apresentados, e a análise estatística foi elaborada no *software* *Statistical Package for the Social Sciences* , versão 20.1 para *Windows* , atribuindo-se nível de significância de 5% e intervalo de confiança de 95%. O teste de Shapiro-Wilk foi utilizado para testar a normalidade dos dados e o teste *t* foi pareado para verificar diferenças do CAP antes da intervenção e após a intervenção, considerando valor de p menor ou igual a 0,05. 

### Aspectos éticos

 Os aspectos éticos referentes à pesquisa com seres humanos foram todos respeitados como determina a resolução 466/12 do Ministério da Saúde e suas complementares ^(^ 2013 ^)^ . A pesquisa foi aprovada pelo Comitê de Ética e Pesquisa do Hospital Universitário da Universidade Federal do Maranhão (protocolo 4.988.517). 

## Resultados

Durante a coleta, 151 puérperas adolescentes realizaram o pré-teste, entretanto, na etapa da aplicação dos pós-teste, houve a perda de 12 participantes, somando 139 puérperas adolescentes na amostra total.

 Os dados antes da intervenção mostraram que o conhecimento, a atitude e a prática foram considerados adequados, com 33,81%, 15,82% e 19,42%, respectivamente, com escore médio entre os três domínios de 32 para “adequado” ( Tabela 1 ). 

**Tabela 1:** - Conhecimento, atitude e prática das puérperas adolescentes antes da intervenção educativa. São Luís e São José de Ribamar, MA, Brasil, 2023

CAP*	**n**	**DP** ^†^	**Mínima**	**Máxima**	**Escore**		**Total**
					**Adequado** **n (%)**	**Inadequado** **n (%)**	
Conhecimento	139	2,68	-	29	47 (33,81)	92 (66,18)	139
Atitude	139	3,14	01	28	22 (15,82)	117 (84,17)	139
Prática	139	4,97	-	31	27 (19,42)	112 (80,57)	139
Média CAP*					32	107	139

* CAP = Conhecimento, Atitude e Prática

^†^ DP = Desvio-padrão

 Após a intervenção, observou-se aumento entre os três domínios CAP. Houve diminuição da média de adequado para 51, entretanto, quando se analisou isoladamente o domínio atitude, observou-se aumento no número de participantes com atitudes inadequadas ( Tabela 2 ). 

 Na associação do CAP adequado, com as variáveis sociodemográficas e gineco-obstétricas, antes da intervenção, houve associação estatisticamente significante para as variáveis idade, escolaridade, estar estudando, local de residência, número de gravidez e realização de pré-natal (p=0,05), como se pode ver na Tabela 3 . 

 Já na variável “usou algum método contraceptivo”, só houve associação com o domínio atitude, nas variáveis idade, escolaridade, número de gravidez, se fez pré-natal, local de residência e estar estudando ( Tabela 4 ). 

 Na associação entre os domínios conhecimento, atitude e prática, antes da intervenção, houve associação estatisticamente significante entre a atitude e a prática (p<0,05). Após a intervenção, houve associação significante entre os três domínios: a prática com o conhecimento (p<0,05) e a atitude (p= 0,05) ( Tabela 5 ). 

**Tabela 2:** - Conhecimento, atitude e prática das puérperas adolescentes após intervenção educativa. São Luís e São José de Ribamar, MA, Brasil, 2023

CAP*	**n**	**DP** ^†^	**Mínima**	**Máxima**	**Escore**		**Total**
					**Adequado** **n (%)**	**Inadequado** **n (%)**	
Conhecimento	139	2,59	-	37	90 (64,74)	49 (35,25)	139
Atitude	139	3,18	-	35	13 (9,35)	126 (90,64)	139
Prática	139	3,26	-	36	51 (36,69)	88 (63,30)	139
Média CAP*					51	88	139

* CAP = Conhecimento, Atitude e Prática

^†^ DP = Desvio-padrão

**Tabela 3:** - Associação do conhecimento, da atitude e da prática com variáveis sociodemográficas e gineco-obstétricas de puérperas adolescentes antes da intervenção educativa, por análise de correlação. São Luís e São José de Ribamar, MA, Brasil, 2023

**Variáveis**	**Total**	**Conhecimento adequado**		**Atitude adequada**		**Prática adequada**	
		**n (%)**	**Valor de p**	**n (%)**	**Valor de p**	**n (%)**	**Valor de p**
**Idade (anos)**							
12-13	28	2 (7,1)		2 (7,1)		2 (7,1)	
14-15	51	1 (1,9)		1 (1,9)		2 (3,9)	
16-17	52	6 (11,5)		-		4 (7,6)	
18-19	8	6 (75,0)		6 (75,0)		5 (62,5)	
**Residência, zona**							
Rural	61	5 (8,19)	0,31	4 (6,5)	0,23	5 (8,1)	0,11
Urbana	78	8 (10,25)		7 (10,2)		9 (11,5)	
**Escolaridade**							
Fundamental I completo	2	1 (50,0)	0,05	-	0,05	-	0,05
Fundamental I incompleto	4	3 (75,0)		2 (50,0)		-	
Fundamental II completo	2	-		-		-	
Fundamental II incompleto	58	15 (25,8)		10 (17,2)		8 (13,7)	
Médio completo	31	13 (41,9)		7 (22,5)		5 (16,1)	
Médio incompleto	42	12 (28,5)		9 (21,4)		6 (14,2)	
**Estar estudando**							
Sim	39	12 (30,8)	0,01	9 (23,1)	0,18	9 (23,1)	0,14
Não	100	5 (5,0)		4 (4,0)		7 (7,0)	
**Método contraceptivo**							
Sim	14	5 (35,7)	0,33	6 (42,9)	0,05	6 (42,9)	0,33
Não	125	12 (9,6)		11 (8,8)		21 (16,8)	
**Gravidez**							
1	98	18 (18,4)	0,05	21 (21,4)	0,05	15 (15,3)	0,05
2	27	13 (48,1)		15 (55,6)		12 (44,4)	
3 ou mais	14	3 (42,2)		3 (33,3)		1 (11,1)	
**Fez pré-natal**							
Sim	116	29 (25,0)	0,05	20 (17,2)	0,05	22 (19,0)	0,05
Não	23	8 (34,7)		5 (21,7)		7 (30,4)	

**Tabela 4:** - Associação do conhecimento, da atitude e da prática com variáveis sociodemográficas e gineco-obstétricas de puérperas adolescentes após intervenção educativa, por análise de correlação. São Luís e São José de Ribamar, MA, Brasil, 2023

**Variáveis**	**Total**	**Conhecimento adequado**		**Atitude adequada**		**Prática adequada**	
		**n (%)**	**Valor de p**	**n (%)**	**Valor de p**	**n (%)**	**Valor de p**
**Idade (anos)**							
12-13	28	4 (14,3)		4 (14,3)		4 (14,3)	
14-15	51	3 (5,8)		3 (5,8)		4 (7,8)	
16-17	52	10 (19,2)		2 (3,8)		6 (11,5)	
18-19	8	3 (37,5)		2 (25,0)		1 (12,5)	
**Residência, zona**							
Rural	61	7 (11,5)	0,05	6 (9,8)	0,01	7 (11,5)	0,01
Urbana 2	78	10 (12,8)		9 (11,5)		11 (14,1)	
**Escolaridade**							
Fundamental I 1 completo	2	-	0,05	-	0,05	-	0,05
Fundamental I incompleto	4	2 (50,0)		2 (50,0)		2 (50,0)	
Fundamental II 2 completo	2	-		-		-	
Fundamental II incompleto	58	17 (29,3)		12 (20,7)		10 (17,2)	
Médio completo	31	15 (48,4)		9 (29,0)		7 (22,6)	
Médio incompleto	42	14 (33,3)		11 (26,2)		8 (19,0)	
**Estar estudando**							
Sim	39	14 (35,9)	0,04	11 (28,2)	0,04	9 (23,1)	0,05
Não	100	7 (7,0)		6 (6,0)		7 (7,0)	
**Método contraceptivo**							
Sim	14	6 (42,9)	0,31	7 (50,0)	0,04	7 (50,0)	0,17
Não	125	13 (10,4)		12 (9,6)		22 (17,6)	
**Gravidez**							
1	98	20 (20,4)	0,05	23 (23,5)	0,05	17 (17,3)	0,05
2	27	15 (55,6)		17 (63,0)		14 (51,9)	
3 ou mais	14	7 (50,0)		7 (50,0)		5 (35,7)	
**Fez pré-natal**							
Sim	116	31 (26,7)	0,05	35 (30,1)	0,05	24 (20,7)	0,05
Não	23	10 (43,4)		7 (30,4)		9 (39,1)	

**Tabela 5:** - Associação entre os domínios conhecimento, atitude e prática de puérperas adolescentes antes e após a intervenção educativa. São Luís e São José de Ribamar, MA, Brasil, 2023

	**Adequado n (%)**	**Inadequado** **n (%)**	**Valor de p**
**Prática antes**			
**Conhecimento**			
Adequado	1 (0,71)	11 (7,91)	0,12
Inadequado	3 (2,15)	26 (19,07)	
**Atitude**			
Adequado	1 (0,71)	15 (10,79)	0,04
Inadequado	2 (1,43)	36 (25,89)	
**Prática após**			
**Conhecimento**			
Adequado	21 (15,10)	14 (10,07)	0,04
Inadequado	15 (10,79)	29 (20,86)	
**Atitude**			
Adequado	1 (0,71)	10 (7,19)	0,05
Inadequado	5 (3,59)	17 (12,23)	

## Discussão

 O puerpério é um momento do ciclo gravídico puerperal oportuno para prevenir, identificar e tratar alterações que comprometem a saúde da mulher ^(^ 2022 ^)^ , a exemplo da reincidência da gravidez não planejada na adolescência. 

 O presente estudo mostrou que a distribuição dos escores relacionados às médias dos domínios conhecimento, atitudes e prática quanto ao uso da minipílula antes da intervenção educativa foram baixos, evidenciando o conhecimento inadequado em relação aos métodos contraceptivos e à vulnerabilidade das puérperas adolescentes, dentre eles, a minipílula como método de prevenção de reincidência de gravidez no pós-parto. Um estudo qualitativo realizado na Tailândia com adolescentes, ao discorrer sobre a reincidência de gestação, identificou que as motivações foram: falta de anticoncepcional, ausência de conhecimentos e de consciência sobre os métodos, avaliados por meio das categorias saber como usar, mas não usar, e por pretender usar e não usar ^(^ 2023 ^)^ . 

 Outro estudo nacional realizado em Pernambuco também destaca o preservativo masculino como o mais conhecido, seguido dos contraceptivos orais, mas as participantes não sabiam qual o tipo de pílula utilizar, quando e como utilizá-las ^(^ 2022 ^)^ , tal dado coincide com o presente estudo e demonstra fragilidade na atitude desses adolescentes em colocar seus conhecimentos em prática devido à falta de informação. 

 O domínio atitude da puérpera adolescente, após a intervenção educativa, aumentou o escore médio de inadequação em relação aos outros domínios que se mantiveram inadequados, porém apresentando melhoras. Acredita-se que a intervenção realizada elevou o conhecimento das participantes sobre o uso adequado da minipílula, porém não foi suficiente para a mudança de atitude na realização de uma prática adequada. É importante complementar o cuidado com intervenções educacionais de modo a melhorar o conhecimento, conforme observado em estudo que utilizou um vídeo educativo, na Tailândia, como complemento ao método tradicional de aconselhamento em consultório, o qual aumentou significativamente o conhecimento sobre o método anticoncepcional e, consequentemente, contribuiu para a preferência pelos métodos apresentados ^(^ 2022 ^)^ . 

 Ao analisar a associação entre as variáveis sociodemográficas com o conhecimento, a atitude e a prática adequadas das puérperas antes da intervenção educativa, verificou-se que as variáveis idade e escolaridade apresentaram associação significativa nos três domínios. Em outras palavras, no domínio conhecimento, tem-se que a maior faixa etária associada à variável “estar estudando” são indicadores que favorecem a capacidade para compreender e melhorar o conhecimento, a atitude e a prática adequada das participantes. Após a intervenção, percebeu-se que, além da idade e da escolaridade, outras variáveis estudadas – como o local de residência – tiveram associações significativas nos três domínios. Nesse contexto, certamente, o uso do álbum seriado, na forma impressa, mediado pela enfermeira na relação interacional com as participantes foi muito importante, configurando uma tecnologia educacional que serve para facilitar a aquisição do CAP das puérperas adolescentes sobre o uso do método contraceptivo em questão ^(^ 2020 ^)^ . 

 A pesquisa voltada ao conhecimento, atitudes e práticas contraceptivas entre adolescentes revelou lacunas que necessitam de atenção para prevenir gravidez não planejada na adolescência, as quais ocorrem frequentemente no pós-parto e podem acontecer no puerpério tardio (11º a 45º dia) ^(^ 2023 ^)^ . Ressalta-se, ainda, a importância da orientação dos pais acerca da contracepção antes do início da vida sexual, bem como o papel dos profissionais de saúde, para discutir, de forma proativa, a contracepção por meio da comunicação individualizada e confidencial ^(^ 2023 ^)^ . 

 Estudos nacionais e internacionais evidenciaram a associação da reincidência da gravidez na adolescência com características socioeconômicas e demográficas da população; isto é, mulheres solteiras; de baixa renda; com escolaridade inadequada para a idade e dificuldade de acesso às ações de planejamento reprodutivo, especialmente no período pós-parto, reiteram as desigualdades sociais existentes entre a população. Nesse contexto, destaca-se o acesso desigual da população feminina ao planejamento reprodutivo ^(^ 2022 ^)^ . Cabe destacar que a gravidez na adolescência está associada ao aumento de complicações maternas e fetais e também é responsável pelo aumento da incidência de abortos inseguros e da mortalidade materna. Assim, é importante conscientizar sobre os métodos eficazes de contracepção pós-parto para prevenir a gravidez nessa fase da vida ^(^ 2022 ^)^ . 

Na análise da associação entre as variáveis gineco-obstétricas com o conhecimento, a atitude e a prática adequadas das puérperas, antes da intervenção educativa, verificou-se que as variáveis “número de gravidez” e “ter feito pré-natal” apresentaram associação significativa nos três domínios. Após a intervenção, além das duas variáveis mencionadas, o uso de método contraceptivo teve associação significativa no domínio atitude. Esse resultado mostra que a intervenção educativa foi profícua, no sentido de possibilitar a compreensão sobre o método, favorecendo a adesão à prática do uso adequado da minipílula.

 Os resultados de outro estudo realizado com adolescentes e mulheres jovens em Guiné revelaram fatores individuais e comunitários associados à necessidade não atendida de contracepção. Os autores sugerem que, além das campanhas de educação sexual e conscientização por meio da mídia e dos locais onde as mulheres se reúnem, o aconselhamento individual em serviços de saúde deve ser melhor direcionado para esse público, considerando suas características individuais e contextuais ^(^ 2023 ^)^ . 

 Tais indicadores, associados com os fatores socioeconômicos, provavelmente influenciaram positivamente no despertar da atenção da puérpera para aprimorar seu conhecimento, principalmente sua atitude para uma prática adequada do uso da minipílula como método contraceptivo no pós-parto ^(^ 2019 ^)^ . 

 Ao se analisar a articulação entre os três domínios, verificou-se associação significativa na relação da atitude com a prática adequada, sendo possível inferir que a tomada de decisão da participante para a prática do método apresentado se correlaciona com os conhecimentos adquiridos previamente e por suas próprias necessidades de usar algum método preventivo. Dessa forma, é possível afirmar que a prática se torna mais efetiva quando há conhecimento, evidenciando uma relação de dependência entre os três domínios. Em adolescentes africanos, foram observadas barreiras relacionadas às concepções equivocadas sobre os efeitos colaterais da contracepção, principalmente a crença de que esta pode causar infertilidade permanente, além do estigma associado ao seu uso e à atividade sexual antes do casamento, assim como a falta de assistência à saúde voltada às necessidades para o uso de contraceptivos ^(^ 2022 ^)^ . 

 Neste estudo, o conhecimento do uso da minipílula como método contraceptivo no pós-parto para a puérpera adolescente tornou-se mais consistente por ter sido mediado pela enfermeira e pelo uso do álbum seriado. Como uma tecnologia educacional, o álbum é uma ferramenta essencial no processo de educação e saúde, em que se almeja um ensino criativo e que desperte o senso crítico do público-alvo ^(^ 2022 ^)^ . O uso de tecnologia educacional favorece a melhoria do conhecimento evidenciado em estudo realizado com adolescentes tailandeses utilizando vídeos educativos para aconselhamento, os quais demonstraram efetividade para melhorar o conhecimento contraceptivo em adolescentes pós-parto ^(^ 2016 ^)^ . 

 Em adolescentes norte-americanas, identificou-se que a tomada de decisões contraceptivas foi influenciada pelas redes sociais e pela comunidade, incluindo os pais e os amigos. As mães desempenharam papel fundamental na transição dos adolescentes para ganharem mais autonomia sobre suas decisões reprodutivas. Os provedores devem apresentar consistentemente aos adolescentes opções contraceptivas abrangentes como um componente dos cuidados de saúde preventivos ^(^ 2022 ^)^ . 

 Participantes do estudo qualitativo realizado na Tailândia indicaram falta de conhecimento sobre contracepção. Embora conheçam os métodos, muitos não têm consciência sobre eles. Além disso, apesar de saberem usá-los, não os utilizam, mesmo expressando a intenção de fazê-lo. Nesse sentido, as usuárias necessitam de aconselhamento na tomada de decisão para escolher o método contraceptivo mais adequado e, assim, evitar gravidezes não planejadas ^(^ 2022 ^)^ . 

 Para o aconselhamento, vale recorrer às diversas alternativas para a prevenção de gravidez na adolescência, como as tecnologias educacionais, capazes de potencializar a promoção, a assistência e o cuidado com a saúde, que se tornam úteis na perspectiva de alcançar as adolescentes de forma mais assertiva. Aqui, destaca-se o álbum seriado, recurso didático, atraente e motivador que pode facilitar a construção do conhecimento pelas puérperas adolescentes sobre o uso adequado da minipílula anticoncepcional no pós-parto ^(^ 2020 ^)^ . 

 Nesse sentido, cabe ao profissional de enfermagem intervir na vivência e nas necessidades de cada adolescente durante a consulta puerperal, não se limitando somente aos protocolos estabelecidos ^(^ 2022 ^)^ . 

Destaca-se como limitação do estudo a dificuldade em estabelecer contato telefônico com as participantes para aplicação do pós-teste, embora a pesquisadora tenha utilizado a estratégia de realizar três tentativas para manter contato. Destaca-se, ainda, a desvantagem atribuída ao desenho quase experimental pelo potencial de generalização reduzido, com resultados menos conclusivos.

O estudo traz contribuição para a enfermagem na discussão do papel do enfermeiro na orientação sexual do adolescente relacionada aos métodos contraceptivos, contribuindo para a prevenção da gravidez não planejada ou para a reincidência, tão comum no período da adolescência. Portanto, o enfermeiro, inserido nesse contexto, tem papel de educador, ou seja, sua atuação também se dá nas práticas educativas e contribui para a prevenção de ISTs, a exemplo da HIV, tanto quanto colabora na prevenção de gravidez não panejada, entre outras necessidades do grupo de adolescentes.

## Conclusão

As puérperas adolescentes apresentaram, antes da intervenção educativa, conhecimentos, atitudes e práticas inadequadas quanto ao uso da minipílula como método de contracepção pós-parto. Após a intervenção, elas apresentaram melhora significativa nos domínios conhecimento e prática, porém a média do domínio atitude, considerado inadequado, aumentou.

Na associação dos domínios com as variáveis sociodemográficas e gineco-obstétricas antes da intervenção, detectou-se associação estatisticamente significante com as variáveis idade, escolaridade, estar estudando, número de gravidez e pré-natal. Após a intervenção, além das variáveis mencionadas, o local de residência, o conhecimento, a atitude e a prática também apresentaram relevância. A idade, a escolaridade, o local da residência, o número de gravidez, ter feito pré-natal e o uso do método contraceptivo foram fatores que influenciaram no uso adequado da minipílula pelas puérperas adolescentes.

Evidencia-se que a tecnologia empregada para a intervenção educativa é efetiva para promoção do conhecimento, da atitude e da prática na prevenção da gravidez e na sua reincidência, usando-se a minipílula como contraceptivo indicado no pós-parto. Entretanto, é necessária sua associação com outras tecnologias, bem como com a rede de apoio familiar e social.

As puérperas adolescentes têm conhecimento, porém ele não é suficiente para a tomada de decisão de uma prática adequada, provavelmente pela precocidade da idade ou pela falta da rede de apoio familiar e social.
